# Supplementary material for: AAV-mediated GBA1 and GDNF rescue neurological defects in a murine model of neuronopathic Gaucher disease
Source: Mol Ther Nucleic Acids. 2025 Mar 7;36(2):102506. doi: 10.1016/j.omtn.2025.102506 (PMC11979523; doi:10.1016/j.omtn.2025.102506)
Supplement: Document S1. Figures S1–S7, Tables S1 and S2 [file mmc1.pdf]

OMTN, Volume *36*

Supplemental information

AAV-mediated GBA1 and GDNF rescue neurological defects in a murine model of neuronopathic Gaucher disease

Yuan Meng, Jiale Zhang, Ruoyue Fan, Wei Pang, Wanyang Zeng, Qingguo Guo, Xuefei Han, Ying Liu, and Guangzuo Luo

Table S1 The antibody used in study

Antibody name	Catalog	Application
anti-Rabbit MAP2	abcam ab32127	IF 1:100
anti-Rabbit LAMP1	Servicebio GB112949	IHC 1:500
anti-Rabbit LAMP1	abcam ab24170	WB 1:1000<br>IF 1:400
anti-Rabbit LC3B	NOVUS NB600-1384	WB 1:1000<br>IHC 1:500
anti-Rabbit α-syn(S129)	abcam ab51253	WB 1:1000
anti-Rabbit CD68	Servicebio GB113109	IHC 1:500
anti-Rabbit CD68	Immunoway YT5210	WB 1:1000
anti-Rabbit GFAP	Servicebio GB11096	WB 1:1000<br>IHC 1:500
anti-Rabbit IBAI	CST 17198T	WB 1:1000
anti-Rabbit IL6	Immunoway YT5348	WB 1:1000
anti-Rabbit IL1β	Immunoway YT5201	WB 1:1000
anti-Rabbit TNFα	Immunoway YT4689	WB 1:1000
anti-Rabbit MAP2	abcam ab5392	WB 1:1000
anti-Rabbit Nurr1	boster PB0332	WB 1:1000
anti-Rabbit TH	proteintech 25859-1-AP	WB 1:1000<br>IHC 1:500
anti-Rabbit NeuN	Servicebio GB11138	WB 1:1000
anti-Rabbit GDNF	abcam ab18956	WB 1:1000
anti-Rabbit β-actin	proteintech 20536-1-AP	WB 1:1000
anti-Rabbit Caspase 3	boster A00334-1	WB 1:1000
anti-Rabbit Bax	boster BA0315-2	WB 1:1000
anti-Rabbit Bcl-2	boster A00040-1	WB 1:1000
anti-Rabbit AKT	Cell Signaling Technology 4691	WB 1:1000
anti-Rabbit pAKT	Cell Signaling Technology 4060	WB 1:1000
anti-Rabbit pGSK3β	Cell Signaling Technology 5558T	WB 1:1000
anti-Rabbit Synapsin-1	Cell Signaling Technology 5297	WB 1:1000
anti-Rabbit α-syn	Cell Signaling Technology 4179	WB 1:1000<br>IF 1:400
anti-Rabbit Caspase 9	Cell Signaling Technology 9508T	WB 1:1000
anti-Rabbit Glucocerebrosidase	Sigma-Aldrich G4171	WB 1:1000

Antibody name	Catalog	Application
anti-Rabbit GFP	Proteintech 50430-2-AP	WB 1:5000
anti-Rabbit GAPDH	Proteintech 10494-1-AP	WB 1:5000
HRP-conjugated Goat Anti-Rabbit IgG(H+L)	Proteintech SA00001-2	WB 1:5000
CoraLite488-conjugated Goat Anti-Rabbit IgG(H+L)	Proteintech SA00013-2	IF 1:1000

Table S2 The Sequence used in RT-PCR

Gene name	Sequence (5'->3')
m*Gfap*-F	GCAGACCTCACAGACGTTGCT
m*Gfap*-R	AGGCTGGTTTCTCGGATCTGG
m*Tnfα*-F	CGTCAGCCGATTTGCTATCT
m*Tnfα*-R	CGGACTCCGCAAAGTCTAAG
m*Inos*-F	CCTCCTTTGCCTCTCACTCTTC
m*Inos*-R	AGTATTAGAGCGGTGGCATGGT
m*Il6*-F	ATGGATGCTACCAAACTGGAT
m*Il6*-R	TGAAGGACTCTGGCTTTGTCT
m*Gdnf*-F	AACATGCCTGGCCTACTTTG
m*Gdnf*-R	GACTTGGGTTTGGGCTATGA
m*Bdnf*-F	GTGACAGTATTAGCGAGTGGG
m*Bdnf*-R	GGGTAGTTCGGCATTGC
m*Nt3*-F	GGTCAGAATTCCAGCCGATGA
m*Nt3*-R	GGCACACACACAGGAAGTGTC
m*Nurr1*-F	CTACGACGTCAAGCCACCTT
m*Nurr1*-R	GGCATCATCTCCTCGGACTG
m*Igf1*-F	TGCTCTTCAGTTCGTGTG
m*Igf1*-R	ACATCTCCAGTCTCCTCAG
m*Igf2*-F	ACAACTTCGATTTGAACCACATTC
m*Igf2*-R	GAGAGCTCAAACCATGCAAACT
m*Gapdh*-F	AGGTCGGTGTGAACGGATTTG
m*Gapdh*-R	TGTAGACCATGTAGTTGAGGT
m*Ccl12*-F	CACGTCCGGAAGCTGAAGAG
m*Ccl12*-R	AGTATGGTCCTGAAGATCACAGC
m*Ccl2*-F	CCACTCACCTGCTGCTACTC
m*Ccl2*-R	AGCTTGGTGACAAAAACTACAGC
m*Cxcl*-F	CCCAAACCGAAGTCATAGCCA
m*Cxcl*-R	CTCCGTTACTTGGGGACACC

Gene name	Sequence (5'->3')
m*Cyba*-F	ACCATGGAGCGATGGTTGT
m*Cyba*-R	AATGGGAGTCCACTGCTCAC
m*Cybb*-F	TTTGTCAAGTGCCCCAAGGT
m*Cybb*-R	ACGCCTATTGTGGTGTTAGGG
m*Cd68*-F	ACGATGACACCTACAGCCAC
m*Cd68*-R	ACAGATATGCCCCAAGCCTTT
m*Sting1*-F	GTCCTGCTAGGTGTCCACTG
m*Sting1*-R	GGAGTATGGCATCAGCAGCC
m*Tnfaip3*-F	AGGGAACTGCCCAGTCTGTA
m*Tnfaip3*-R	TCGTCATTCCAGTTCCGAGT
m*IRF7*-F	GAGCTTGGATCTACTGTGGGC
m*IRF7*-R	CGGCCCTTGTACATGATGGT
m*OAS3*-F	GACCGCTCTAGCATCTCCAC
m*OAS3*-R	TATCGAGTGACAACCTGGCG
m*CASP12*-F	GGCCTGCAGAGGCAGATATAAT
m*CASP12*-R	GCATCTGGGTCAGTTCACCT
m*NAIP2*-F	ACTCACAGATGCGCAGTGAA
m*NAIP2*-R	TCACTTGTGGTTTCCATGGCT
m*NAIP5*-F	TGATTGAAATTGCCAGGGCG
m*NAIP5*-R	CACACATTGACCCAGAGCCT
m*NLRP3*-F	AGCCAGAGTGGAATGACACG
m*NLRP3*-R	TCACCTCTCGGCAGTGGATA
m*CTSB*-F	TGACTGGGGTGATAATGGCTTC
m*CTSB*-R	GAAACTGCAGCACAGAACCG
m*CTSD*-F	GACATCCACTACGGCTCAGG
m*CTSD*-R	TCCACCCTGCGATACCTTGA
m*CTSE*-F	ACTACATCCTGCCGGACTTG
m*CTSE*-R	GTGGGGTGCACTCTTATGCT
m*LGMN*-F	TGGGCTGGAGTAAGTAAGTCTTC
m*LGMN*-R	TTGACGCTGTACCAGTCACC
m*SLC11A1*-F	TGGCTGGGTTCAAACTCCTC
m*SLC11A1*-R	TAGTAGAACTCTGCCCCGCT
m*Gba1*-F	AGGCAGGGCTAGAGAGA
m*Gba1*-R	GAATCTACAGTGGGAAGT

Figure S1

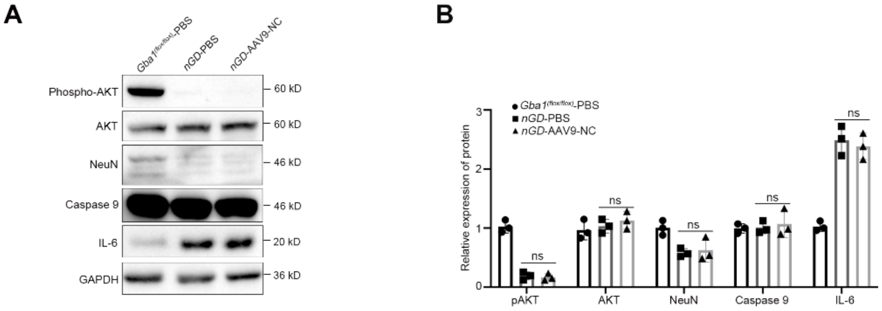

Figure S1. AAV9-NC does not affect *nGD* mouse model.

(A) One month post-delivery, western blot analysis of the whole-brain of *Gba1*$^{(flox/flox)}$-PBS, *nGD*-PBS, and *nGD*-AAV9-NC groups.

(B) Statistical quantitative diagram of (A). ns, no significance.

Figure S2

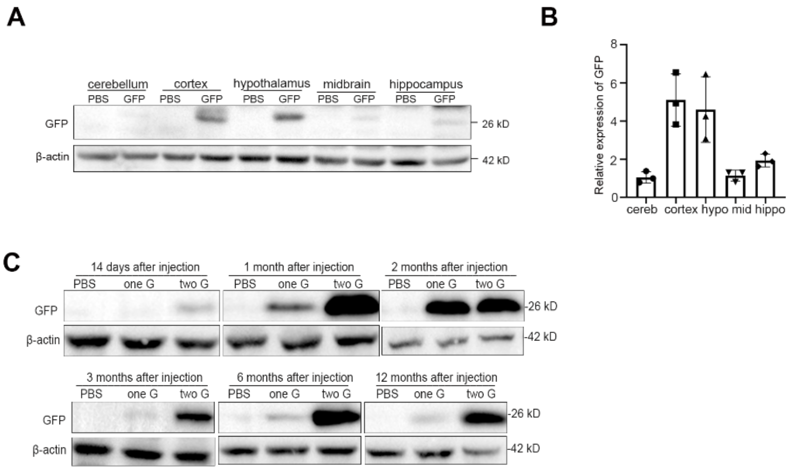

Figure S2. Distribution of AAV9-GFP in the brain of newborn mice after intraparenchymal administration.
(A) Western blot analysis of five brain reigons in C57BL/6 mice one month after AAV9-GFP injection. (B) Statistical quantitative diagram of (A). (C) The dose and time dependence of intraparenchymal administration were detected in C57BL/6 whole-brain.

Figure S3

A

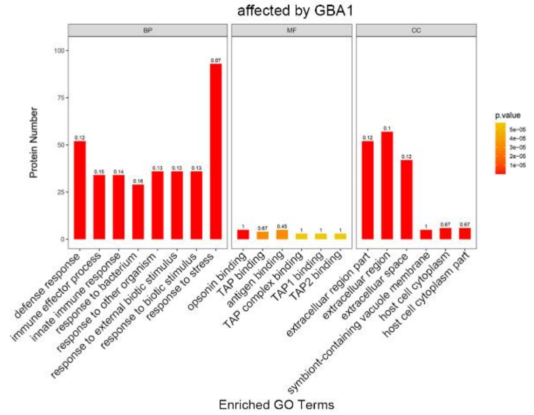

B

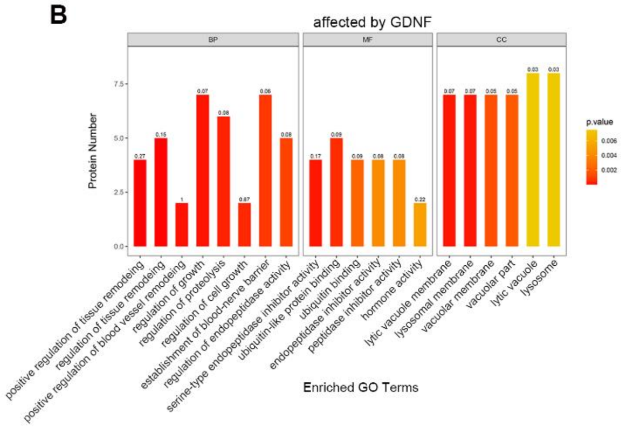

Figure S3. Proteomic detection of *nGD* mice after treatment.

(A) GO functional annotation analysis of proteome sequencing after GBA1 treatment for three months.

(B) GO functional annotation analysis of proteome sequencing after GDNF treatment for three months.

GO, Gene Ontology; BP, Biological Process; MF, Molecular Function; CC, Cellular Compoment.

Figure S4

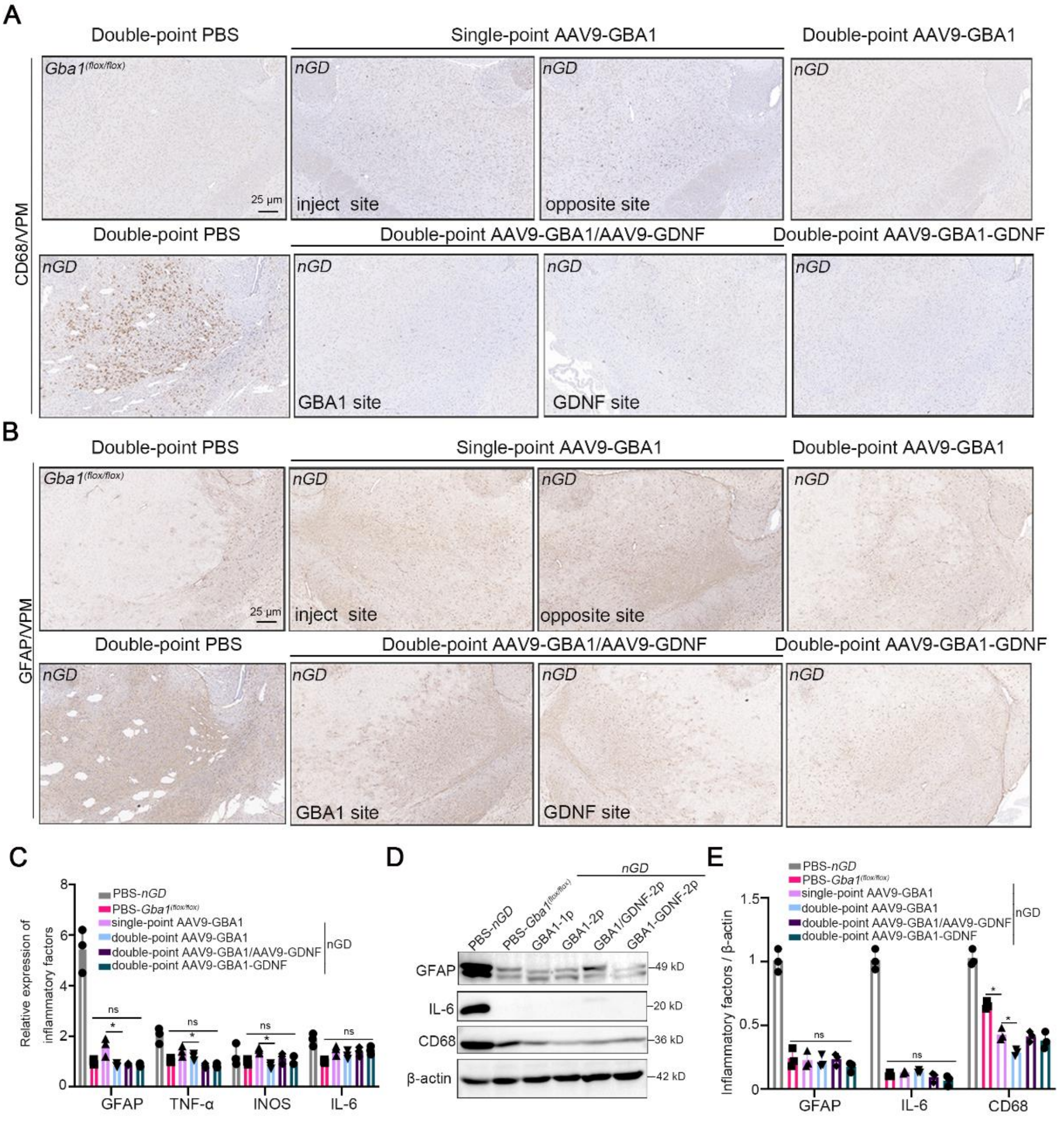

Figure S4. Intracerebral inflammation detection in the *nGD* mice after treatment.

(A) and (B) Three months after treatment, CD68 and GFAP staining was performed in the VPM regions of the mice *Gba1*$^{(flox/flox)}$, *nGD* and *nGD* treatment groups. (C) The mRNA relative expression of inflammatory factors after homogenization in the whole-brain tissue of mice in *Gba1*$^{(flox/flox)}$, *nGD* and *nGD* treatment groups by $2^{-\Delta\Delta CT}$. (D) Western blot was performed to detect the expression levels of inflammation-related proteins in the *Gba1*$^{(flox/flox)}$, *nGD* and *nGD* treatment groups. (E) Quantitative statistical graph of (D). All data are expressed as mean ± standard deviation. The one-way ANOVA method was used for the analysis. Tukey's method was used for multigroup comparisons (n = 3 per group). * $P < 0.05$, ** $P < 0.01$. CD68, Cluster of Differentiation 68. GFAP, Glial fibrillary acidic protein.VPM, ventral posteromedial thalamic nuclei.

Figure S5

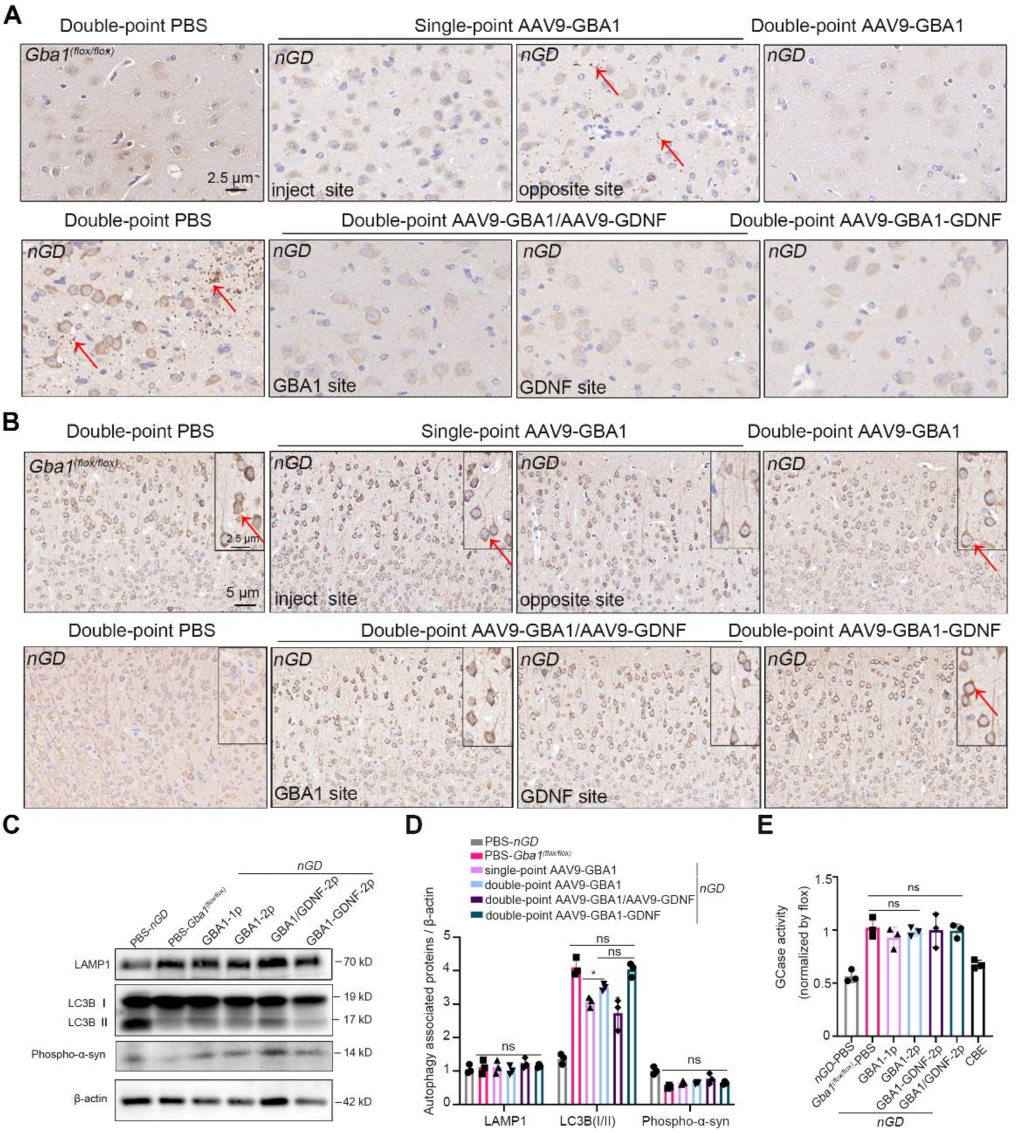

Figure S5. Detecting lysosome function in the *nGD* mice after treatment.

(A) Three months after treatment, LAMP1 staining of lysosomal membrane proteins in VPM sections of mice with *Gba1$^{(flox/flox)}$*, *nGD* and *nGD* treatment groups, with an arrow pointing to positive staining. (B) LC3B staining was performed on sections of the hippocampal S1BF area of mice with *Gba1$^{(flox/flox)}$*, *nGD* and *nGD* treatment groups. The small box indicates an enlarged image of the positive area, and the arrow indicates positive staining. (C) Expression levels of autophagy-related proteins in the *Gba1$^{(flox/flox)}$*, *nGD* and *nGD* treatment groups were detected using Western blot. (D) The statistical quantitative graph of (C). (E) Detection of enzyme activity in mice in the *Gba1$^{(flox/flox)}$*, *nGD* and *nGD* treatment groups after treatment for three months. All data are expressed as mean ± standard deviation. The one-way ANOVA method was used for the analysis. Tukey's method was used for multigroup comparisons (n = 3 per group). * $P < 0.05$. VPM, ventral posteromedial thalamic nuclei, SIBF, the primary sensory barrel field.

Figure S6

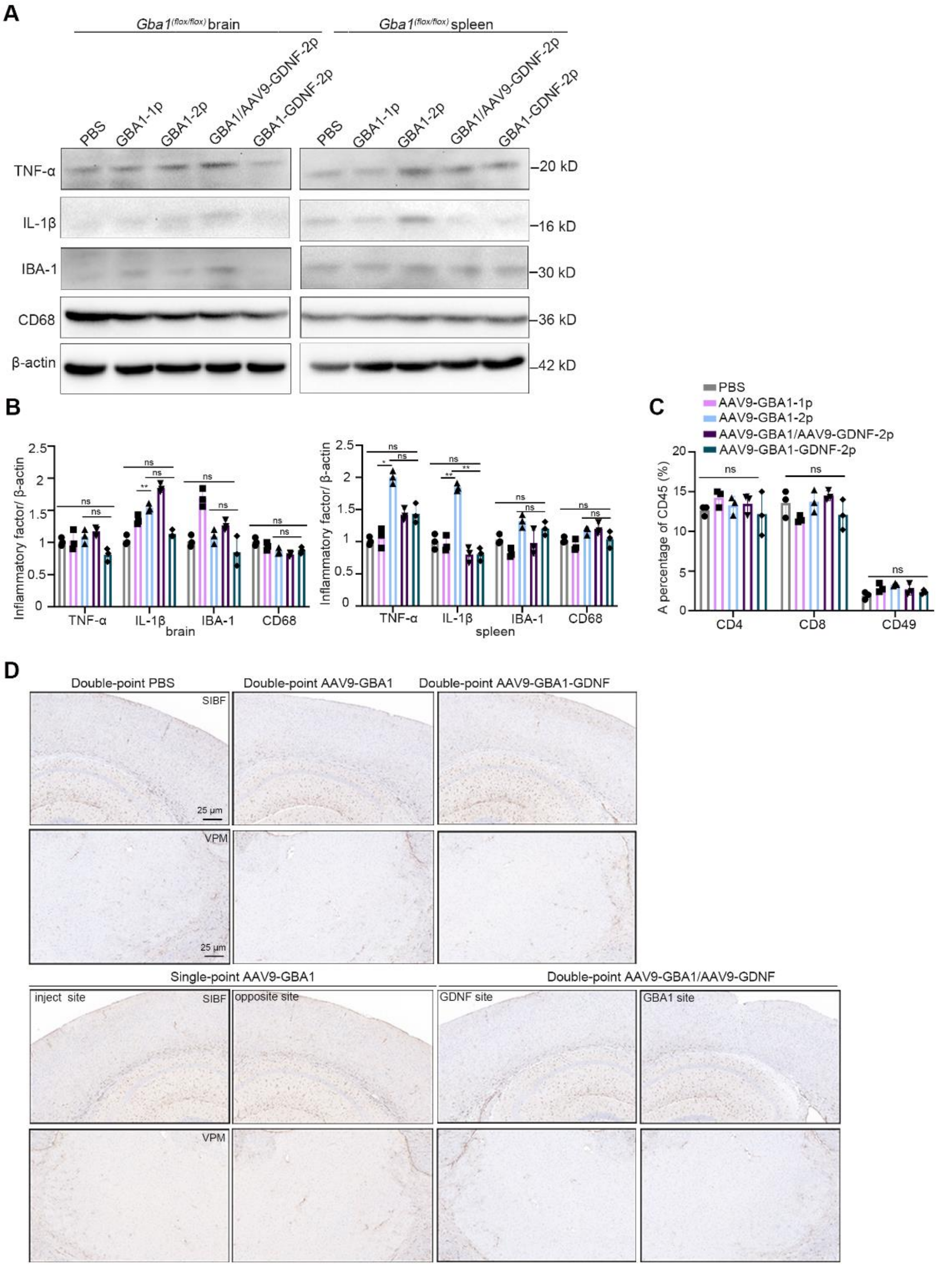

Figure S6. Safety evaluation of intraparenchymal administration of *Gba1*$^{(flox/flox)}$ mice.

(A) Western blot was performed to detect the expression of inflammation-related proteins in *Gba1*$^{(flox/flox)}$ mice 4 months post-intraparenchymal delivery. (B) Diagram of quantitative analysis of A. (C) Flow cytometry was used to detect the percentages of CD4$^+$T cells, CD8$^+$T cells, and CD49$^+$T cells among CD45$^+$T cells in *Gba1*$^{(flox/flox)}$ mice 4 months post-intraparenchymal delivery. (D) Positive areas for GFAP were observed in the S1BF and VPM brain sections from *Gba1*$^{(flox/flox)}$ mice 4 months post-intraparenchymal delivery. All data are expressed as mean ± standard deviation. The one-way ANOVA method was used for the analysis. Tukey's method was used for multigroup comparisons (n = 3 per group). * $P < 0.05$, ** $P < 0.01$. SIBF, the primary sensory barrel field. VPM, ventral posteromedial thalamic nuclei.

Figure S7

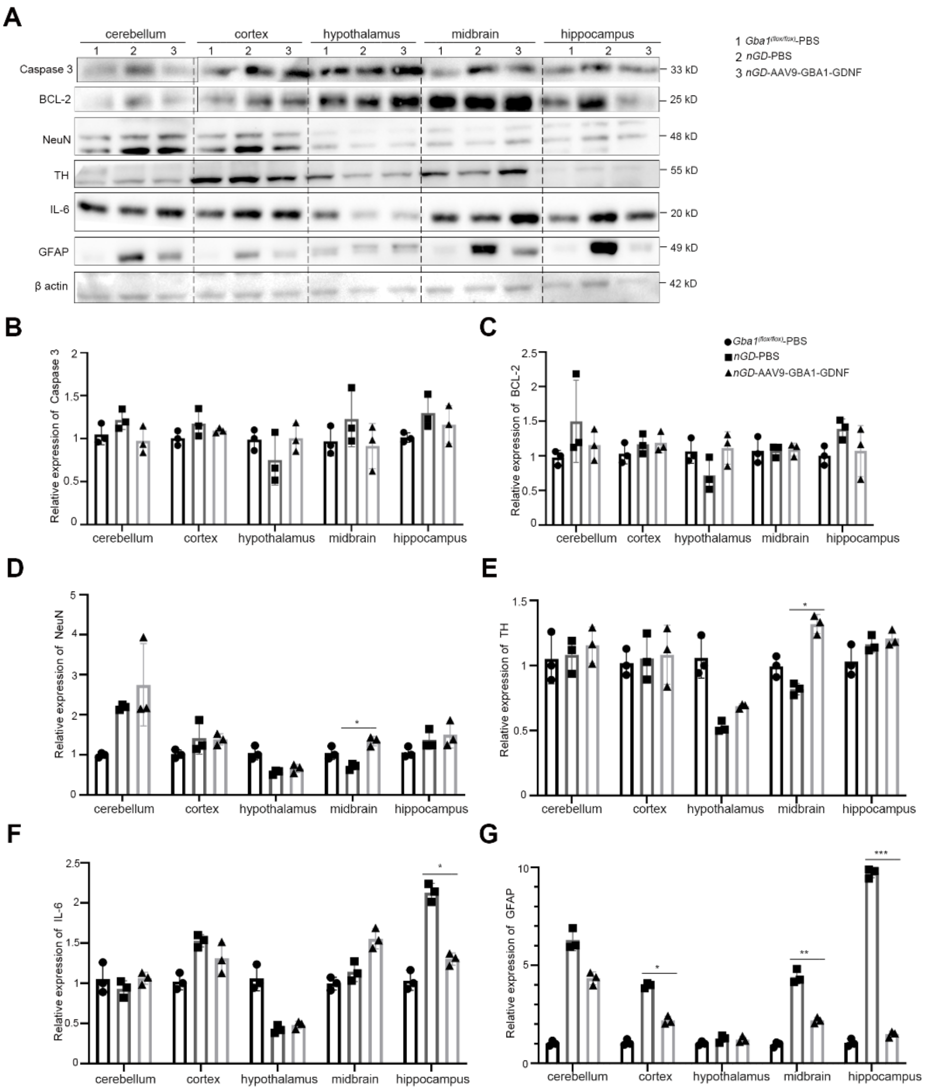

Figure S7. Effect of AAV9-GBA1-GDNF in different brain regions of *nGD* mice.

(A) Western blotting analysis showing the protein level in the cerebellum, cortex , hypothalamus, midbrain and hippocampus of *Gba1*$^{(flox/flox)}$, *nGD*, and *nGD*-AAV9-GBA1-GDNF mice. (B-G) Statistical quantitative diagram of (A). * $P < 0.05$, ** $P < 0.01$.
