# Supplementary material for: AAV-mediated GBA1 and GDNF rescue neurological defects in a murine model of neuronopathic Gaucher disease
Source: Mol Ther Nucleic Acids. 2025 Mar 7;36(2):102506. doi: 10.1016/j.omtn.2025.102506 (PMC11979523; doi:10.1016/j.omtn.2025.102506)
Supplement: Document S2. Article plus supplemental information [file mmc2.pdf]

Molecular Therapy
Nucleic Acids
Original Article

AAV-mediated GBA1 and GDNF rescue neurological defects in a murine model of neuronopathic Gaucher disease

Yuan Meng,[1] Jiale Zhang,[1] Ruoyue Fan,[3] Wei Pang,[2] Wanyang Zeng,[1] Qingguo Guo,[2] Xuefei Han,[3] Ying Liu,[2] and Guangzuo Luo[1,3]

[1]Institute of Health Sciences, China Medical University, Shenyang 110122, China; [2]Department of Biochemistry and Molecular Biology, China Medical University, Shenyang 110122, China; [3]Bionce Biotechnology, Ltd., Nanjing 210061, China

Neuropathic Gaucher disease (nGD) is a life-threatening disease that progresses rapidly and is caused by a *glucosylceramidase beta 1* (*GBA1*) mutation, which encodes the lysosomal hydrolase β-glucocerebrosidase (GCase). Nerve damage in nGD, associated with stunted growth and development, arises from the degeneration and death of nervous system cells, which is often irreversible. Approved therapies effectively reduce the substrate burden outside the central nervous system (CNS) through augmenting mutant enzyme activity with pharmacologic recombinant GCase or by inhibiting glucocerebroside synthesis. However, these therapies do not provide neuroprotection. In this study, we developed a novel double-gene therapy based on adeno-associated virus (AAV), AAV9-GBA1-GDNF, which stably expresses human GBA1 and glial derived neurotrophic factor (GDNF) over the long term. Pathological, molecular, and proteomic tests in the *nGD* model confirmed that the early stages of the disease are characterized by GBA1 deficiency, loss of neuronal function, and even neuronal death. After treatment with AAV9-GBA1-GDNF, the lifespan of *nGD* mice was extended, and weight, brain development, and motor ability were recovered. Additionally, GBA1 and GDNF additively prevented irreversible neuronal death by activating the AKT/GSK3β pathway. These findings offer potential therapeutic strategies for *nGD* and other neurodegenerative diseases associated with lysosomal dysfunction.

INTRODUCTION

Gaucher disease (GD), one of the most common lysosomal storage disorders, is divided into three clinical phenotypes according to the degree of neurological involvement.[1] Type 1 GD is clinically non-neuronopathic in the central nervous system (CNS) until late middle age or beyond, when a minority of patients and carriers may develop GBA1-associated Parkinson disease (PD).[2] Some adult patients with type 1 GD may develop systemic manifestations, such as severe visceral or bone disease.[3] Unlike type 1 GD, patients with type 2 and type 3 GD exhibit clinically heterogeneous central neurological manifestation. The disease progresses rapidly as cranial nerves become affected.[1,4–8] Types 2 and 3 GD are collectively referred to as neuronopathic GD (nGD), which has been ignored because of its low incidence, acute onset, and poor survival rates. Among these, children with type 2 GD die during the perinatal period or infancy.[3] The *glucosylceramidase beta 1* (*GBA1*) mutation or deletion in nGD is usually concentrated between exons 6 and 10. Common mutations include *K198E*, *V394L*, *Y304C*, *S107L*, *R257Q*, *G202R*, *F213I*, and *L444P*.[9–12] Neuropathological studies demonstrate the presence of Gaucher cells, gliosis, and microglial nodules in type 2 GD brains, eventually leading to neuronal loss.[7,13–15] More importantly, neuronal death is always irreversible, underscoring the critical need to protect neuronal structure and function in a timely manner to prevent neuronal degeneration and death for treating patients with nGD.[16]

There are currently no effective treatments for nGD. The treatment for types 2 and 3 GD has largely been an extension of therapies developed for type 1 GD,[15] such as enzyme replacement therapy (ERT) and substrate reduction therapy (SRT). However, these approaches have significant limitations in treating nGD.[11,17,18] Existing therapies cannot prevent severe neurological damage in patients with nGD, which is a major the cause of death.[19,20] First, expensive therapeutic drugs that are large-molecule proteins with a short half-life require repeated administration, which increases the burden on patients' lives and on the economy.[21,22] Second, the inability of these drugs to efficiently cross the blood-brain barrier (BBB) limits their effectiveness.[23] Last, existing therapy aims to enhance GCase activity, thereby "indirectly" reducing systemic cell death. However, it lacks "direct" protection for neuronal structure and function, which is crucial to prevent the irreversible neuronal death in a timely manner.[24–26] Therefore, development of novel therapeutic approaches to overcome the limitations of type 2 GD therapies is essential.

Received 8 October 2024; accepted 4 March 2025;
https://doi.org/10.1016/j.omtn.2025.102506.

Correspondence: Ying Liu, Department of Biochemistry and Molecular Biology, China Medical University, Shenyang 110122, China.
E-mail: liuying@cmu.edu.cn
Correspondence: Guangzuo Luo, Institute of Health Sciences, China Medical University, Shenyang 110122, China.
E-mail: gzluo@cmu.edu.cn

Published by Elsevier Inc. on behalf of The American Society of Gene and Cell Therapy.
This is an open access article under the CC BY license (http://creativecommons.org/licenses/by/4.0/).

Neurotrophic factors, such as brain-derived neurotrophic factor (BDNF), cerebral dopamine neurotrophic factor (CDNF), neurturin (NRTN), and glial cell line-derived neurotrophic factor (GDNF), regulate neuronal cells by inducing synaptic formation, thus promoting neuronal survival, growth, differentiation, and maturation.[27–29] In addition to these "direct" stimulatory effects on neuronal function and growth, GDNF has been reported to reduce the toxic effects on dopaminergic neurons in PD.[30,31] These studies confirm the positive role of GDNF in protecting the structure, function, and survival of neurons. This suggests that increasing GDNF levels through gene therapy may be a potential option for the timely protection of neurons in type 2 GD.[31–33]

Gene therapy based on adeno-associated virus (AAV) is a potential option for addressing the short-term sustainability of existing therapies. AAV vectors are suitable for the long-term delivery of target genes owing to their ability to achieve sustained expression, high stability, and low immunogenicity.[34,35] Moreover, growing evidence supports the safety and efficacy of AAV9-based gene therapies for treating CNS disorders.[36–39]

To address the limitations of current type 2 GD therapies, we constructed a novel AAV-based dual-gene therapy vector, AAV9-GBA1-GDNF, which can stably express human GBA1 and GDNF. Using the acute nGD mouse model, we examined the long-term efficacy of GBA1 and GDNF in the brain following a single intraparenchymal injection of AAV9-GBA1-GDNF. After confirming that AAV9-GBA1-GDNF could significantly prolong the lifespan and restore the motor abilities in treated mice, the effects of the individual and combined actions of GBA1 and GDNF on brain atrophy and neuronal death in nGD mice were analyzed. Furthermore, proteomic analyses and molecular evaluations were conducted to determine whether AAV9-GBA1-GDNF could play a timely protective role in neuronal structure and function. Finally, the specific signaling pathway involving AAV9-GBA1-GDNF was explored, and primary neurons were extracted for verification.

RESULTS

Severe brain lesions in *nGD* mice

To better study nGD, we crossed *Gba1*$^{(flox/+)}$;*Nestin-Cre* mice with *Gba1*$^{(flox/flox)}$ mice, creating *Gba1*$^{(flox/flox)}$; *Nestin-Cre* mice (referred to as *nGD* mice throughout the text), as previously described (Figure 1A).[40] At 21 days post-birth, *nGD* mice displayed dyskinesia, tail elevation, eating disorders, paralysis, and in some cases, death (Figure 1B). On the day of birth, the offspring were numbered, and their DNA was extracted by using the toe amputation method. Genotypes of the offspring were identified using PCR and agarose gel electrophoresis. As shown in Figure 1C, no. 4 is an *nGD* mouse, with the homozygous *Flox* tag detected at 240 base pairs (bp) and the positive *Nestin-Cre* tag is located at 600 bp. The expression of *GBA1* in the brains of *nGD* mice was detected using RT-qPCR and western blotting. The results showed that *nGD* mice had only 20% of *GBA1* transcript levels compared with those in control *Gba1*$^{(flox/flox)}$ mice. Compared with *Gba1*$^{(flox/flox)}$ mice, GBA1 protein expression was not detected in the brains of *nGD* mice (Figures 1D and 1E). To determine whether *Gba1*$^{(flox/flox)}$ mice could be used as experimental controls, we compared GBA1 protein expression levels among C57BL/6J, *Gba1*$^{(flox/+)}$, and *Gba1*$^{(flox/flox)}$ mice at P21. As shown in Figure 1F, GBA1 expression levels in C57BL/6J, *Gba1*$^{(flox/+)}$, and *Gba1*$^{(flox/flox)}$ mice were almost consistent. Neuropathological analysis of *nGD* brains was performed using immunohistochemical (IHC) staining. We observed neuronal phagocytosis (blue box), diffuse neuronal shrinkage (red arrow), neuronal necrosis (detected by TUNEL staining), and vacuole formation (red box) in *nGD* mice (Figure 1G). Through proteomic analysis of the murine *nGD* model, we explored the effects of *nGD* on protein expression. Gene Ontology (GO) enrichment analysis revealed that the *nGD* model was significantly enriched in metabolic processes in the biological process category. In the cellular components category, the *nGD* model showed significant enrichment in synapse-related proteins and lysosome-related proteins (Figure 1H).

GDNF significantly promotes synaptic formation and neuron survival *in vitro*

Conduritol B epoxide (CBE), a GCase inhibitor, was used to treat SH-SY5Y cells to construct a cell model of *nGD*.[41,42] Additionally, HT22 cells treated with 1-methyl-4-phenyl-1,2,3,6-tetrahydropyridine (MPTP) were used to mimic neuronal death in *nGD*. Based on these two models, the effects of AAV9-GDNF on synaptogenesis and neuronal death were evaluated *in vitro*. As shown in Figure 2A, almost no synaptogenesis was observed in CBE-treated SH-SY5Y cells. After treatment with AAV9-BDNF, AAV9-CDNF, AAV9-GDNF, or AAV9-NRTN, the length of the synapses in SH-SY5Y cells increased significantly. Compared with other neurotrophic factors, GDNF had a more significant effect on synaptic length (Figure 2B). Examination of GCase activity showed that enzyme activity in SH-SY5Y cells was decreased by 25 μM CBE, and neurotrophic factors did not restore GCase activity (Figure 2C). Expression of mature neuron marker MAP2 in SH-SY5Y and *nGD* mouse-derived primary hippocampal neurons was detected by immunocytochemical staining. MAP2 expression was increased both in SH-SY5Y and *nGD* hippocampal neurons treated with AAV9-GDNF compared with that in untreated neurons, suggesting that GDNF promoted synaptogenesis in the *nGD* model (Figure 2D). The effect of AAV9-GDNF on cell viability was detected by CCK-8. The results showed that the viability of HT22 and SH-SY5Y cells decreased with increasing MPTP concentrations, with HT22 cells being more sensitive to the toxic effects of MPTP than SH-SY5Y cells. Therefore, 2.25 mM MPTP and AAV9-GDNF were selected to co-treat HT22 cells to detect changes in cell viability. The CCK-8 assay results showed that cell viability in the co-treated group was significantly higher than that of in the MPTP-treated group (Figures 2E and 2F).

AAV9-GBA1-GDNF improves brain atrophy, lifespan, and motor deficits in *nGD* mice

Gba1$^{(flox/flox)}$; *Nestin-Cre* mice, which exhibit rapid motor dysfunction associated with severe neurodegeneration and apoptotic cell death within the brain, were used as *nGD* models.[40] As shown in

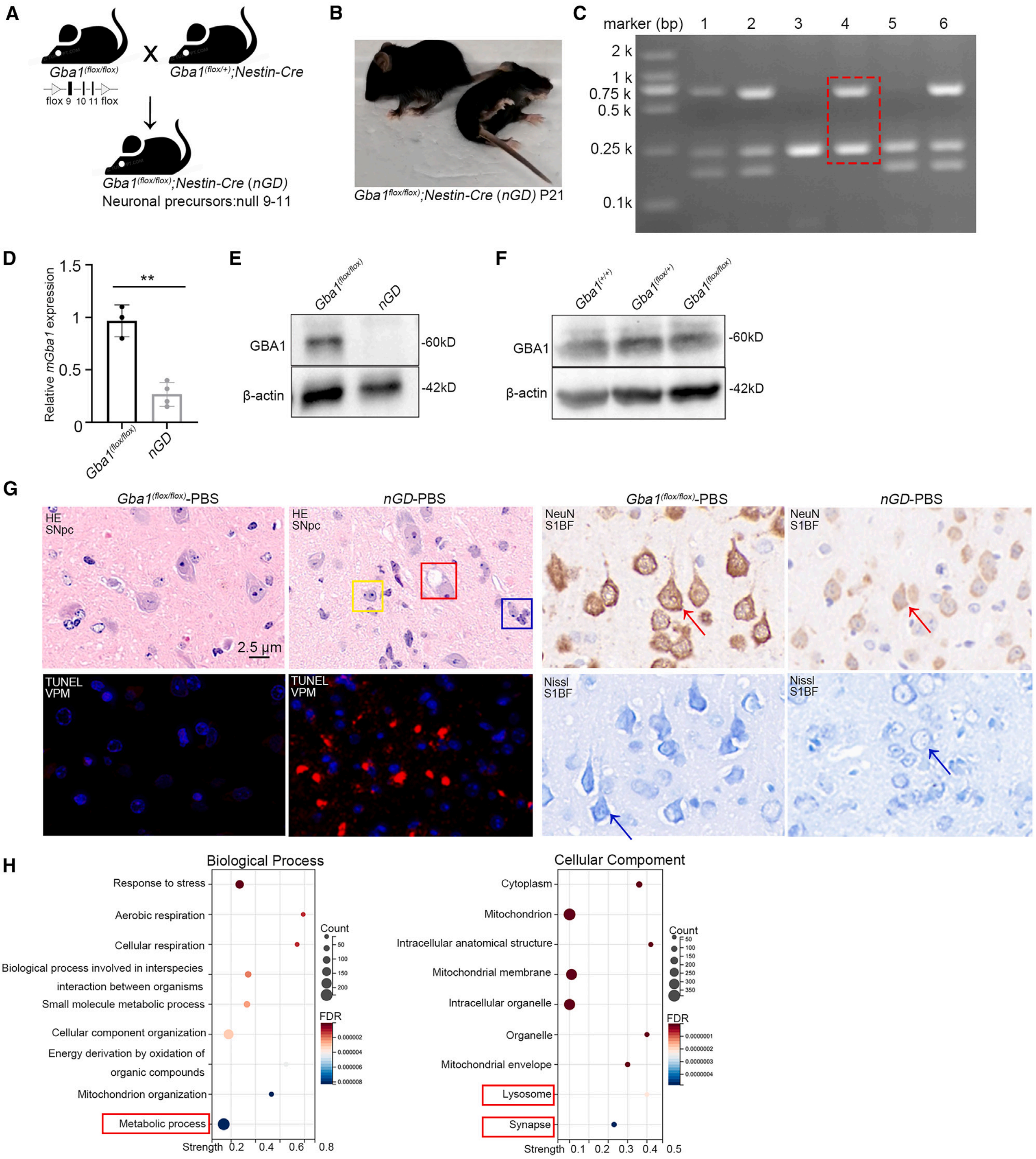

Figure 1. Neuropathological changes in *nGD* mice

(A) Schematic view of the generation of *nGD* mice, *Gba1*$^{(flox/flox)}$ mice with exons 9–11 being flanked by two *loxP* sites and being deleted in the central nervous system upon mating with the *Gba1*$^{(flox/+)}$; *Nestin-Cre* mice. (B) The morphology of *nGD* mice at P21. (C) Identification of *nGD* mice genotype by agarose gel electrophoresis. (D) The expression of *GBA1* molecule and (E) GBA1 protein level of *nGD* and *Gba1*$^{(flox/flox)}$ mice brain. (F) Western blot analysis of GBA1 protein in the brains of *Gba1*$^{(+/+)}$, *Gba1*$^{(flox/+)}$, and *Gba1*$^{(flox/flox)}$ mice. (G) Sections of brain tissue from *nGD* and *Gba1*$^{(flox/flox)}$ mice. (H) Biological process and cellular component GO functional annotation analysis of *nGD* and *Gba1*$^{(flox/flox)}$ mice. All data are expressed as mean ± standard deviation. The one-way ANOVA method was used for the analysis. Tukey's method was used for multigroup comparisons (n = 3 per group). **$p < 0.01$.

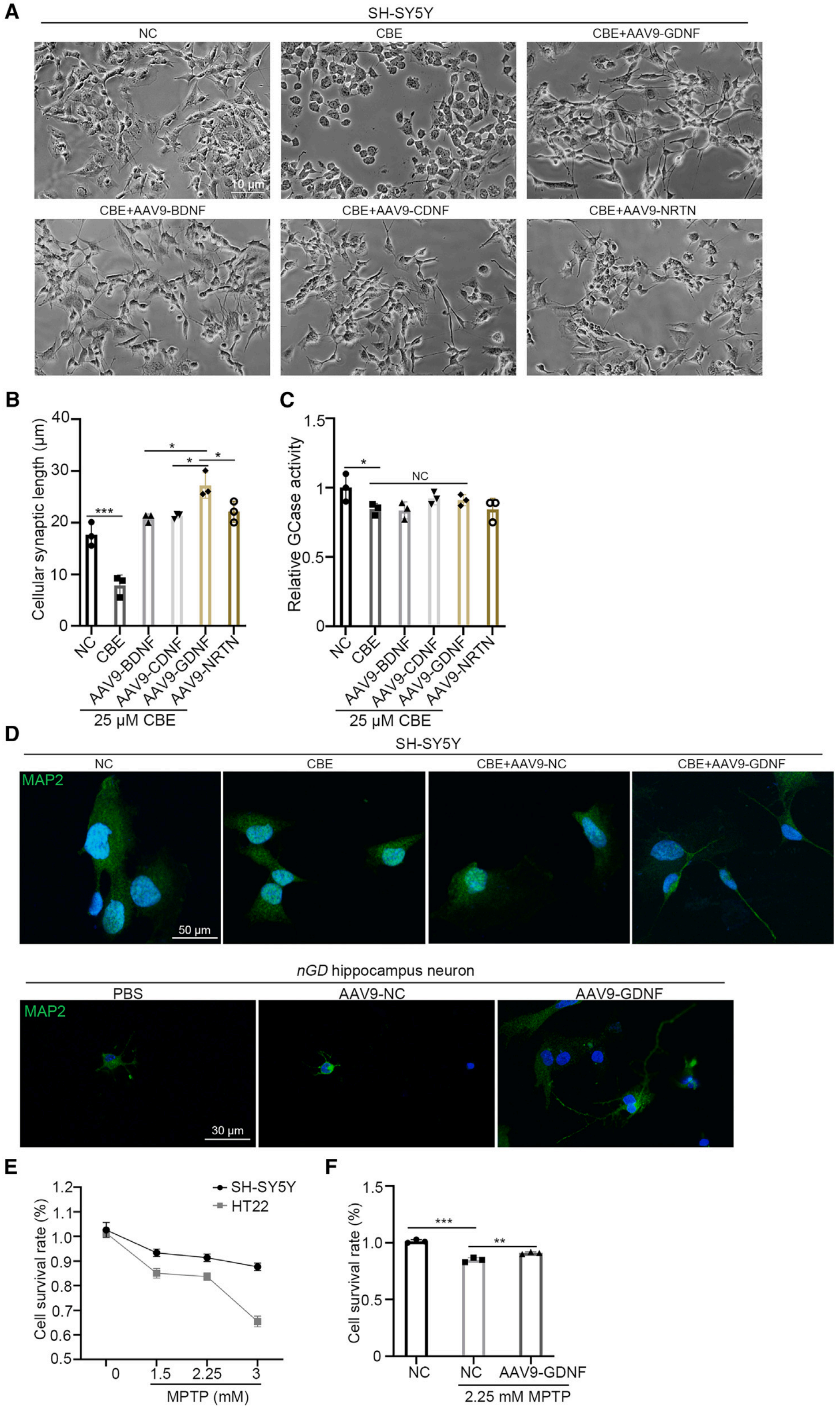

(legend on next page)

Figure 3A, phosphate-buffered saline (PBS), AAV9-NC, AAV9-GBA1, AAV9-GDNF, or AAV9-GBA1-GDNF were delivered to the brains of *nGD* mice at P1 via intraparenchymal delivery. In the *nGD* treatment group, single-point (1e10 vg/μL, 2 μL into one cerebral hemisphere) and double-point (1e10 vg/μL, 2 μL into both cerebral hemispheres) administration of AAV9-GBA1 were used to test the diffusion of the drug in the brain and the dose dependence of the treatment. AAV9-NC was used as a viral vector control to confirm that the virus did not affect disease progression (Figure S1). AAV9-GFP was used as a tracker to detect drug distribution via intraparenchymal delivery (Figure S2).

To explore the effects of AAV9-GBA1-GDNF on brain development in *nGD* mice, magnetic resonance imaging (MRI) scans were performed on the brains of mice 3 months post-delivery. Significant brain atrophy was observed in mice in the single-point AAV9-GBA1 and the double-point AAV9-GBA1 groups, compared with that in $Gba1^{(flox/flox)}$ mice. Remarkably, no significant difference in brain size was observed between the AAV9-GBA1/AAV9-GDNF and double-point AAV9-GBA1-GDNF groups, compared with that in the $Gba1^{(flox/flox)}$ mice, confirming the beneficial effects of GDNF on brain atrophy in *nGD* mice (Figures 3B and 3C).

As previously reported, *nGD* mice exhibited weight loss and a limited lifespan, dying at approximately P21 after birth.[40] After treatment, the mice lived significantly longer than those in the *nGD* group and showed no significant differences in body weight compared with those in the $Gba1^{(flox/flox)}$ group (Figures 3D and 3E). The motor and coordination abilities of the mice were further analyzed by using the rotating rod, tension, and balance beam tests (Figures 3F–3H). Compared with $Gba1^{(flox/flox)}$ mice, there were no significant differences in the motor and coordination abilities of mice in the double-point AAV9-GBA1 or AAV9-GBA1-GDNF groups, demonstrating improved motor deficits in *nGD* mice.

Proteome sequencing revealed the potential additive effects of AAV9-GBA1-GDNF

Proteomic sequencing was conducted on the brain tissue of mice in different experimental groups, including the $Gba1^{(flox/flox)}$ (3 months), *nGD* (P21), double-point AAV9-GBA1 (3 months), and double-point AAV9-GBA1-GDNF (3 months) groups. This study investigated the potential mechanism of action of AAV9-GBA1-GDNF in *nGD* mice. Four-dimensional label-free quantitative proteomics was used to analyze the number and function of differentially expressed proteins (DEPs) after protein quantitation. Proteins with a fold change ≤ 0.5 were considered downregulated DEPs, while those with a fold change ≥ 1.5 were regarded as upregulated DEPs. Compared with DEPs in the $Gba1^{(flox/flox)}$ group, 199 upregulated and 250 downregulated DEPs were observed in the *nGD* group, which were considered disease-related DEPs. Compared with the DEPs in the *nGD* group, 276 upregulated and 217 downregulated DEPs were observed in the AAV9-GBA1 group. Among these, 138 upregulated DEPs in the AAV9-GBA1 group were members of the downregulated disease-related DEPs, which had conflicting expression patterns. In total, 283 DEPs in the AAV9-GBA1 group had conflicting expression patterns and were considered GBA1-related DEPs. Similarly, 308 DEPs in the AAV9-GBA1-GDNF group were considered GBA1-GDNF-related DEPs. Subsequently, GBA1-related and GBA1-GDNF-related DEPs were analyzed using Venn diagrams (Figure 4A). The intersection of GBA1-GDNF-related and GBA1-related DEPs (233 proteins) represents protein expression normalized to GBA1 in *nGD* mice. The complement of GBA1-GDNF-related DEPs (75 proteins) represents protein expression normalized to that of GDNF in *nGD* mice. Kyoto Encyclopedia of Genes and Genomes (KEGG) enrichment of the intersection and complement were analyzed, respectively. In KEGG analysis, the intersection was markedly enriched in the lysosomal membrane, lytic vacuole membrane, and lysosome (Figure 4B), while the complement was enriched in the mTOR, PI3K-AKT, and glutamatergic synapses (Figure 4C). GO functional annotation analysis of proteome sequencing after treatment for 3 months is shown in Figures S3A and S3B.

AAV9-GBA1-GDNF maintains neuronal function in *nGD* mice

We further investigated the effects of AAV9-GBA1-GDNF on neuronal function by performing Nissl staining in *nGD* mice. Brain sections of *nGD* mice were obtained at P21, and those of the other groups were obtained 3 months after treatment. Almost no Nissl bodies were observed in the cerebral cortex (layer V) of mice in the *nGD*, with emerging vacuoles and swelling. Compared with the *nGD*, basophilic Nissl bodies were observed in the unilateral AAV9-GDNF and double-point AAV9-GBA1-GDNF groups (Figure 5A). Tyrosine hydroxylase (TH) a key enzyme in dopamine synthesis, is typically used to mark dopaminergic neurons. TH staining in the substantia nigra pars compacta (SNpc) showed an increased number of TH-positive neurons in the AAV9-GDNF and AAV9-GBA1-GDNF groups compared with those in *nGD* (Figure 5B). We speculate that GDNF contributes to cortical and TH neuronal recovery in *nGD* mice. RNA and proteins were extracted from the whole brains of *nGD* mice to explore changes in neurotrophic factors. Compared with the *nGD* mice, AAV9-GBA1-GDNF promoted the expression of neurotrophic factors, including BDNF, neurotrophic factor 3 (NT3), nuclear receptor-associated protein (NURR1), and insulin-like growth factors (IGF1/IGF2) (Figure 5C). The detection of protein levels demonstrated the stimulatory effect of

Figure 2. The role of neurotrophic factors in *in vitro* conditions

(A) Addition of the inhibitor CBE to the SH-SY5Y cells and synaptogenesis induced by the neurotrophic factor. (B) Measurement of SH-SY5Y synaptic length. (C) Measurement of SH-SY5Y GCase activity. (D) SH-SY5Y and *nGD* hippocampal neuron treated by PBS or AAV9-GDNF. (E) CCK8 detected the survival rate of SH-SY5Y and HT22 cells after MPTP treatment. (F) Survival rates of untreated, MPTP-treated, MPTP, and GDNF-treated HT22 cells were measured using CCK8. All data are expressed as mean ± standard deviation. The one-way ANOVA method was used for the analysis. Tukey's method was used for multigroup comparisons (n = 3 per group). *$p < 0.05$, **$p < 0.01$, ***$p < 0.001$.

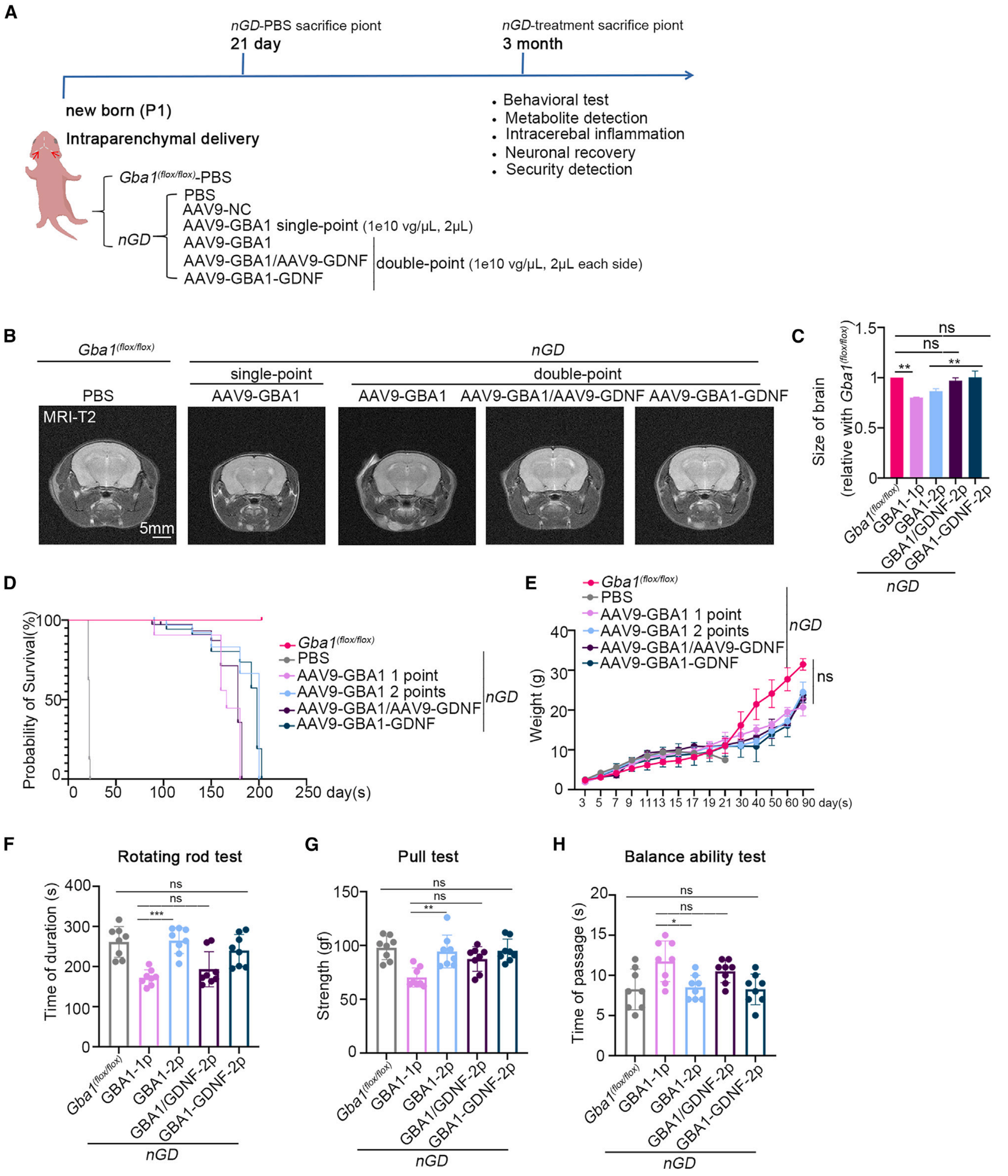

(legend on next page)

AAV9-GBA1-GDNF on the secretion of MAP2, NURR1, and NeuN compared with the AAV9-GBA1 group (Figures 5D and 5E).

AAV9-GBA1-GDNF suppresses neuro-inflammation and cell apoptosis in *nGD* mice

Accumulation of substrates caused by *GBA1* mutations in the brain leads to an inflammatory response. Astrocytes and microglia, brain-residing glial cells, protect neurons by secreting various neurotrophic factors and removing dead neurons, toxic debris, and abnormal proteins through phagocytosis and degradation.[43–45] After treating the *nGD* mice, we examined the recovery of glial cells and found that both single and double-target treatment could restore intracranial inflammatory responses (Figure S4). Cellular inflammation can cause nerve cell death, and TUNEL staining can detect DNA breaks. Therefore, we used tissue immunofluorescence to detect apoptosis caused by neuronal degeneration in *nGD* mice. Extensive TUNEL signaling was observed in the hippocampus and ventral posteromedial thalamic nuclei (VPM) regions of brain tissue in *nGD* mice at P21 (Figures 6A and 6B). After treatment with AAV9-GBA1-GDNF, there was a significant reduction in TUNEL-positive staining compared with that in the other groups (Figure 6C). Compared with the upregulated apoptotic proteins, including Caspase 3, Caspase 9, BAX, and BCL-2 in the brains of *nGD* mice, the expression of Caspase 3 in the AAV9-GBA1-GDNF group was significantly more suppressed than that in the AAV9-GBA1 group (Figures 6D and 6E).

AAV9-GBA1-GDNF activates the AKT/GSK-3β signaling pathway

To verify the reliability and credibility of the proteomic sequencing, we detected the expression of selected DEPs using RT-qPCR and western blotting. As shown in Figures 7A and 7B, after double-point AAV9-GBA1 treatment, there was a significant downregulation of chemokines and apoptosis-related molecules at the transcriptional level in *nGD* mice, comparable to that in *Gba1*$^{(flox/flox)}$ mice. Western blot analysis showed that pAKT/AKT levels in the AAV9-GBA1-GDNF group were higher than those in the AAV9-GBA1 group. Glycogen synthase kinase 3 (GSK3), phosphorylated GSK3 affects a variety of biological activities such as cell proliferation, growth, and survival. Our result showed that pGSK-3β levels in the AAV9-GBA1-GDNF group were significantly decreased compared with those in AAV9-GBA1 group. The above results suggested that GDNF might strengthen the inhibitory effect of GBA1 on apoptosis by activating the AKT/GSK-3β pathway. Also, there was significantly downregulated expression of Caspase 3 in the AAV9-GBA1-GDNF group compared with that in the AAV9-GBA1 group. Interestingly, there was no significant difference in expression of the apoptosis regulator BAX between the AAV9-GBA1-GDNF and AAV9-GBA1 groups. In addition, there was a remarkable upregulated expression of Synapsin1 in the AAV9-GBA1-GDNF group compared with the AAV9-GBA1 group, suggesting that GDNF may regulate axons and promote synaptogenesis in the *nGD* model (Figures 7C and 7D). To explore the potential effects of AAV9-GBA1-GDNF, we used an inhibitor of AKT/GSK-3β (Laduviglusib)-treated HT22 cells. The results showed that the pathway was not significantly activated when the cells were treated with inhibitor alone or when co-treated with AAV9-GBA1-GDNF (Figures 7E and 7F).

AAV9-GBA1-GDNF suppressed neuronal apoptosis in *nGD* primary hippocampal neurons

To confirm the additive protective effects of AAV9-GBA1-GDNF on neurons in *nGD* mice, we extracted primary hippocampal neurons from newborn *nGD* mice. On day 1 after isolation, primary hippocampal neurons were treated with PBS, AAV9-GBA1, or AAV9-GBA1-GDNF (1e10 vg) (Figure 8A). The expression of TUNEL, α-syn, and lysosome-associated membrane protein1 (LAMP1) of primary neurons was detected by immunofluorescence staining. The result showed the abnormal expression of TUNEL-positive signals, and α-syn was significantly suppressed after AAV9-GBA1 treatment. Compared with AAV9-GBA1, AAV9-GBA1-GDNF showed a stronger inhibitory effect on the expression of TUNEL-positive signals, indicating that GDNF enhanced the anti-apoptotic effect of GBA1 (Figures 8B and 8C). On the other hand, there was no significant difference between the AAV9-GBA1 group and the AAV9-GBA1-GDNF group on the expression of α-syn and LAMP1, indicating that GDNF alone had no significant effect on lysosomal function and lysosomal-associated autophagy in this *in vitro* model, which may be due to differences between primary neurons *in vitro* and the brain microenvironment *in vivo* (Figures 8D–8G).

AAV9-GBA1-GDNF promoted the recovery of lysosomal accumulation in *nGD* mice

To further detect the expression of LAMP1, we performed IHC on mouse brains. Representative LAMP1 immuno-stained images showed positive-LAMP1 around or evenly distributed outside the cell membrane (Figure S5A, red arrows) in the VPM region, suggesting lysosomal swelling and rupture caused by metabolic abnormalities in the brains of *nGD* mice. In the AAV9-GBA1/AAV9-GDNF group, LAMP1 expression was mainly distributed around the cell membrane at the injection site without lysosomal swelling or rupture, indicating that GDNF plays a role in lysosomal metabolism. Similarly, no LAMP1 expression outside the cell membrane was observed in the brains of the double-point AAV9-GBA1 and double-point AAV9-GBA1-GDNF groups indicating the rescue of lysosomal function in

Figure 3. Behavioral assessment of the *nGD* mice at 3 months post-delivery

(A) Flow diagram of administration. (B) Images of the T2-weighted maximum cross-section of the brains of mice in the MRI. The treatment group and the control group at 3 months post-delivery. (C) Quantitative statistical graph of (B). (D) and (E) Survival curves and weight monitoring of mice in the *Gba1*$^{(flox/flox)}$, *nGD*, and *nGD* treatment groups. (F), (G), and (H) The running time (s), value of the tension tester (gf), passing time of mice in the *Gba1*$^{(flox/flox)}$ and *nGD* treatment groups. All data are expressed as mean ± standard deviation. The one-way ANOVA method was used for the analysis. Tukey's method was used for multigroup comparisons (n = 8 per group). *$p < 0.05$, **$p < 0.01$, ***$p < 0.001$.

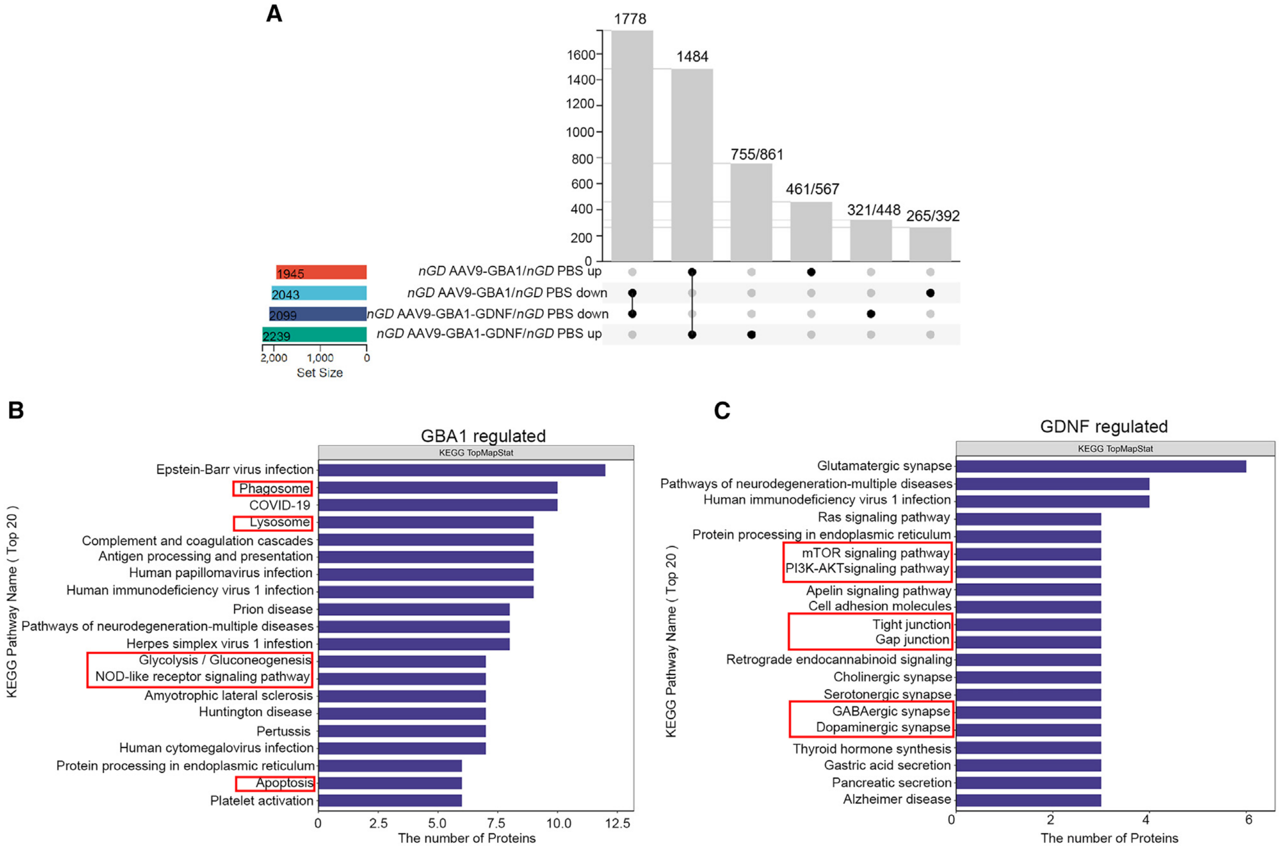

Figure 4. Proteomic analysis of the brain tissue after treatment in the *nGD* mice

(A) Three months post-delivery of AAV9-GBA1 and AAV9-GBA1-GDNF, Venn maps related to pathogenic proteins were drawn using proteomic sequencing. (B) KEGG pathway analysis via proteomic sequencing 3 months post-delivery of AAV9-GBA1. (C) KEGG pathway analysis by proteomic sequencing 3 months post-delivery of AAV9-GBA1-GDNF treatment. KEGG, Kyoto Encyclopedia of Genes and Genomes. (n = 3 per group).

nGD mice. Substrate fluorescence assays showed that GCase activity was significantly enhanced after AAV9-GBA1-GDNF treatment (Figure S5E).

Safety evaluation of AAV9-GBA1-GDNF *in vivo*

To evaluate the safety of AAV9-GBA1-GDNF *in vivo*, we assessed the immune and inflammatory responses in the brains and spleens of *Gba1*$^{(flox/flox)}$ mice. *Gba1*$^{(flox/flox)}$ mice were divided into groups at P1 and euthanized at 4 months post-administration. Brain homogenates and spleen tissues were collected. Subsequently, we detected the expression of immune factors tumor necrosis factor (TNF)-α, interleukin (IL)-1β, IBA-1, and CD68 in the brain and spleen by performing western blotting. The expression of TNF-α and IL-1β in the mice with single-point injection was lower than those with double-point injection. No differences were observed in the expression of AAV9-GBA1-GDNF or *Gba1*$^{(flox/flox)}$, indicating the safety of AAV9-GBA1-GDNF (Figures S6A and S6B). To further determine the effect of AAV9-GBA1-GDNF on systemic immune responses, flow cytometry was performed to determine the proportions of $CD4^+$, $CD8^+$, $CD45^+$, and $CD49^+$ T cells (macrophage markers) in the spleen. Results showed no difference in the T cell populations between the AAV9-GBA1-GDNF and *Gba1*$^{(flox/flox)}$ groups (Figure S6C). Simultaneously, GFAP and CD68 (data not shown) expression was detected by IHC. As shown in Figure S6D, there was no obvious astrocyte proliferation in the S1BF and VPM.

DISCUSSION

Clinically, the incidence of GD ranges from 1 in 40,000 to 1 in 60,000, increasing to 1 in 800 in Ashkenazi Jews, patients with nGD accounting for approximately 5% of all patients with GD.[11,46] However, it is important to note that in many Asian countries (Egypt, China, Korea, Japan, India), patients with nGD may represent up to 50% of all GD cases.[26,47,48] Existing GD-related studies have mainly focused on GBA1 functional compensation and substrate accumulation, including eliglustat, ambroxol, and gene therapy methods compensating for GBA1 (Clinicaltrials: NCT04127578, NCT04411654), ignoring neurodegeneration in nGD.[49,50] In this study, we demonstrated that AAV9-GBA1-GDNF can preserve GCase function and effectively protect neuronal cells by activating the AKT/GSK3β signaling pathway after a one-time intraparenchymal injection in a

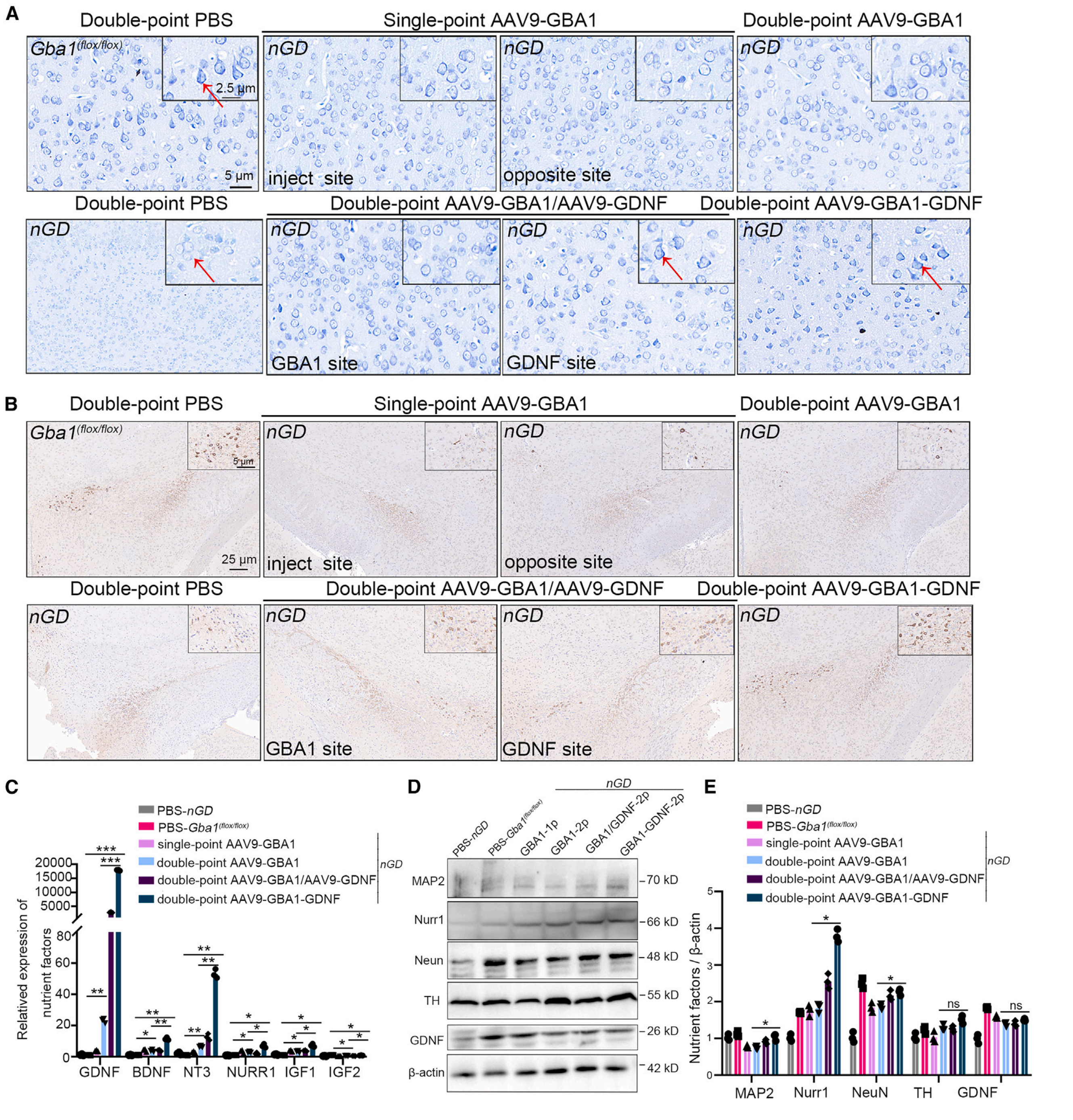

Figure 5. Neuronal recovery in the brain of *nGD* mice after treatment

(A) Three months post-delivery, Nissl staining of the fifth layer of the S1BF section of mice with *Gba1*$^{(flox/flox)}$, *nGD*, and *nGD* treatment groups, is indicated with the small box showing an enlarged image of the selected area. (B) TH staining of the SNpc brain sections of mice in the *Gba1*$^{(flox/flox)}$, *nGD*, and *nGD* treatment groups, the circles represent positive areas. (C) Relative mRNA expression of the nutrient factor by $2^{-\Delta\Delta CT}$ after homogenization in the whole-brain tissue of mice in the *Gba1*$^{(flox/flox)}$, *nGD*, and *nGD* treatment groups. (D) Expression levels of neuron-related proteins in the whole brain of *Gba1*$^{(flox/flox)}$, *nGD*, and *nGD* treatment groups detected using western blot. (E) Statistical quantitative graph of (D). All data are expressed as mean ± standard deviation. The one-way ANOVA method was used for the analysis. Tukey's method was used for multigroup comparisons (n = 3 per group). *$p < 0.05$, **$p < 0.01$, ***$p < 0.001$. TH, tyrosine hydroxylase.

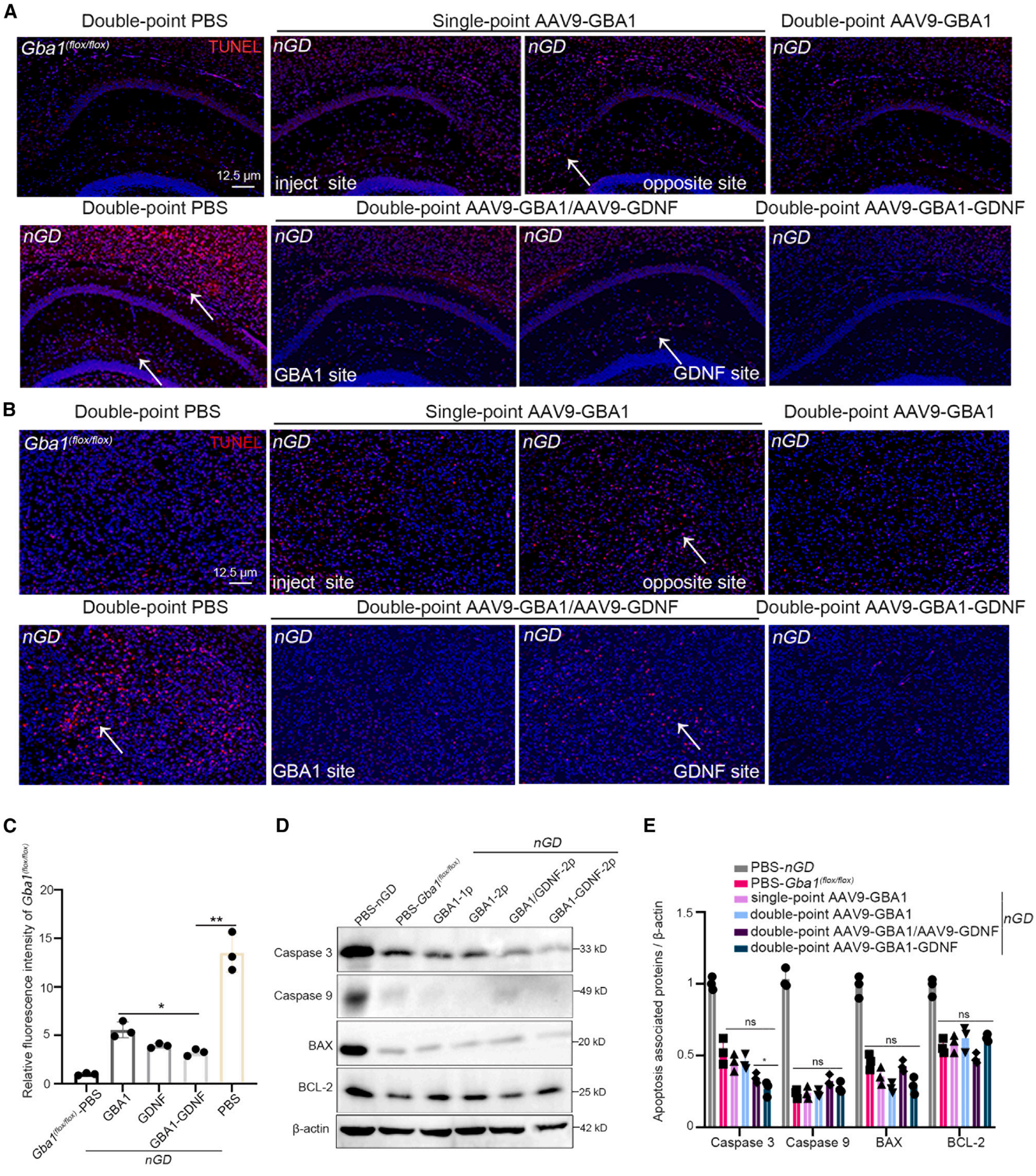

Figure 6. Apoptosis was detected in *nGD* mice after treatment

(A) TUNEL staining was observed in hippocampus and (B) VPM regions of *Gba1*$^{(flox/flox)}$, *nGD*, and *nGD* treatment groups. The positive cells and nuclei were red and blue, respectively. (C) Relative fluorescence intensity of (A) and (B). (D) Expression of apoptosis-related proteins in the *Gba1*$^{(flox/flox)}$, *nGD*, and *nGD* treatment groups was detected using western blot. (E) Statistical quantitative graph of (D). All data are expressed as mean ± standard deviation. The one-way ANOVA method was used for the analysis. Tukey's method was used for multigroup comparisons ($n = 3$ per group). $^*p < 0.05$.

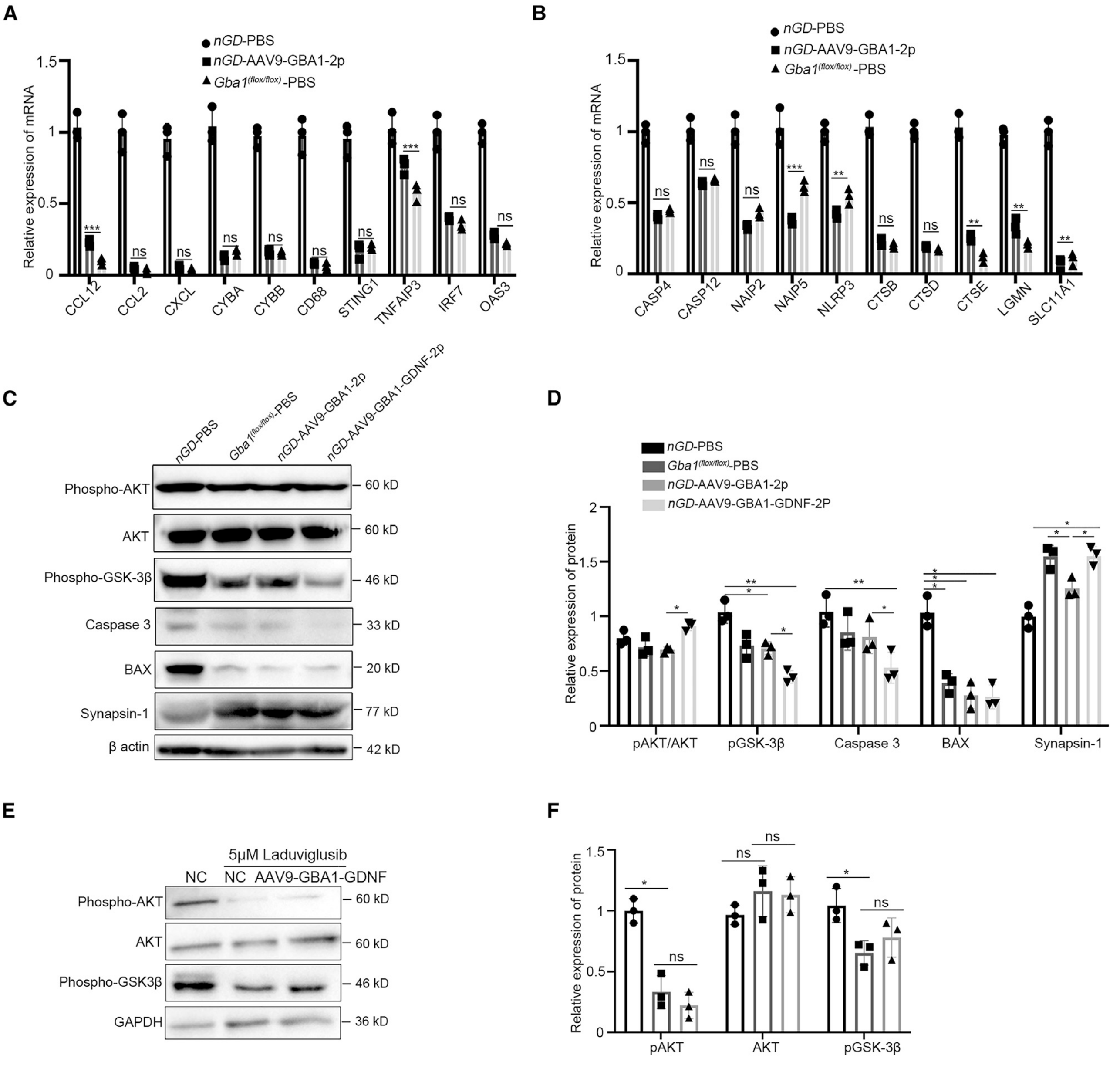

Figure 7. Validation of associated genes by proteomic analysis

(A) Relative expression levels of chemokines related to immune response in mice in PBS-*nGD*, two-point AAV9-GBA1-*nGD*, and PBS-*Gba1*$^{(flox/flox)}$ groups. (B) Relative expression levels of apoptosis-related molecules in PBS-*nGD*, two-point AAV9-GBA1-*nGD*, and PBS-*Gba1*$^{(flox/flox)}$ groups. (C) Western blot analysis was performed to detect pathway-related protein expression levels in PBS-*nGD*, two-point AAV9-GBA1-*nGD*, two-point AAV9-GBA1-GDNF-*nGD*, and PBS-*Gba1*$^{(flox/flox)}$ groups. (D) Statistical quantitative diagram of (C). (E) Western blot analysis was performed to detect untreated, 5 μM Laduviglusib treated, and 5 μM Laduviglusib and AAV9-GBA1-GDNF co-treated HT22 cells. (F) Statistical quantitative diagram of (E). All data are expressed as mean ± standard deviation. The one-way ANOVA method was used for analysis. Tukey's method was used for multigroup comparison (n = 3 per group). *$p < 0.05$, **$p < 0.01$, ***$p < 0.001$.

mouse model of *nGD*, which has the potential to be used in nGD treatment.

Intravenous (i.v.) injection, intracerebroventricular injection (ICV), and intraparenchymal injection were commonly used to treat CNS disease. Compared with intraparenchymal administration, ICV can deliver larger doses and achieve wider drug distribution within brain tissue[51]; however, studies have shown that drugs in the cerebrospinal fluid are preferentially distributed to the systemic blood circulation and rarely penetrate into deep areas of the brain.[52] Intravenous injection is the method of drug administration with the least physical trauma to the patient; however, when i.v. is used for gene therapy, a

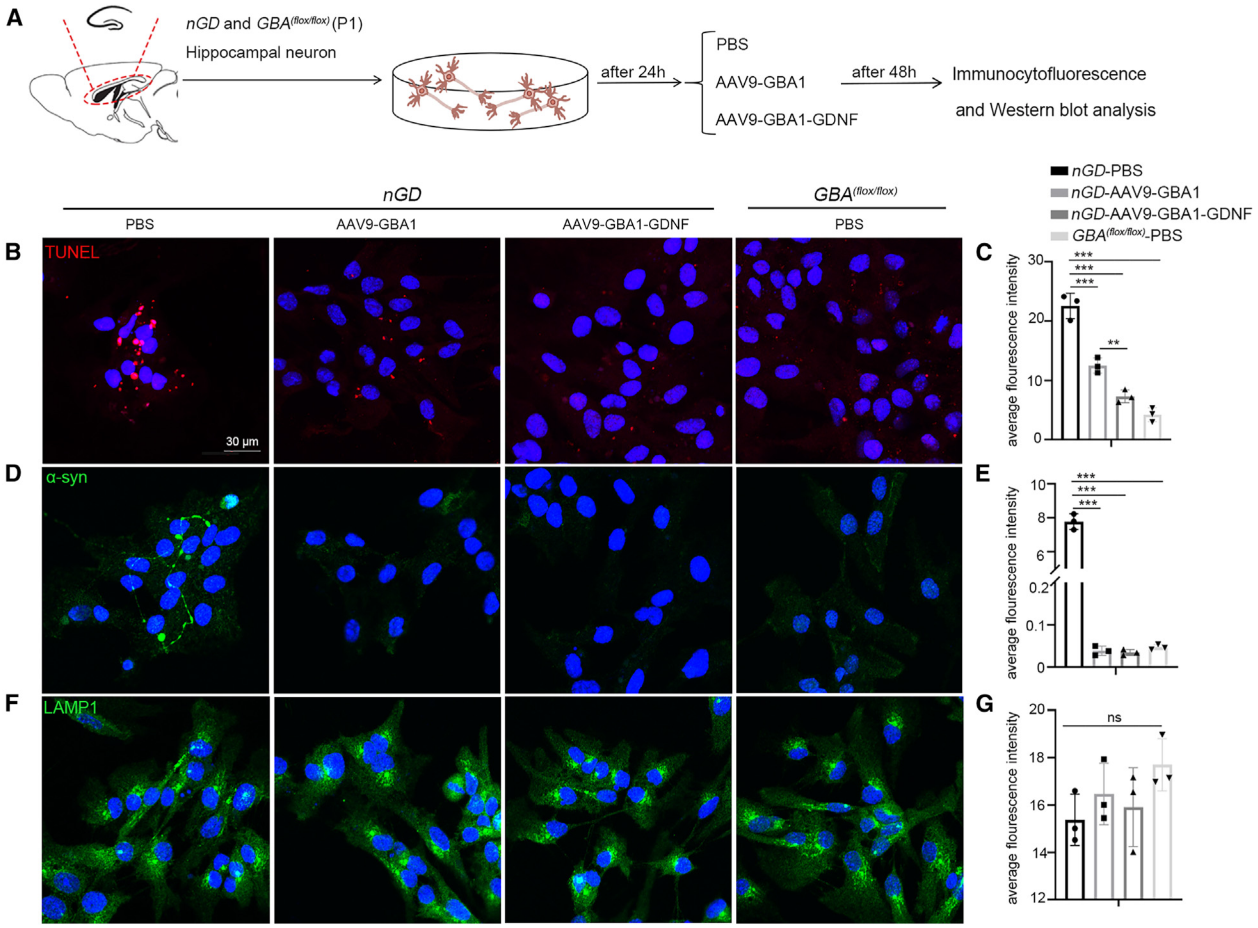

Figure 8. Validation of *nGD* primary hippocampal neurons *in vitro*

(A) Schematic diagram of primary hippocampal neurons processing by *nGD* and *Gba1*$^{(flox/flox)}$. Primary hippocampal neurons were treated with PBS, AAV9-GBA1, or AAV9-GBA1-GDNF, then immunofluorescence staining by (B) and (C) TUNEL, (D) and (E) α-syn, (F) and (G) LAMP1. All data are expressed as mean ± standard deviation. One-way analysis of variance was used for statistical analysis. The Tukey method was used to compare multiple groups (n = 3 per group). $^{**}p < 0.01$, $^{***}p < 0.001$.

large amount of drug is used, which can lead to high costs and immune reactions.[53,54] The intraparenchymal delivery used in this study not only facilitated our comparison of the efficacy of GBA1 and GDNF, but is also a widely used CNS delivery route during the neonatal stage.[55,56] We used GFP as a label to test whether the drug reached most areas of the brain and the persistence of expression (Figure S6).

In the present study, *Gba1*$^{(flox/flox)}$; *Nestin-Cre* was used as a pathological model because it can accurately simulate nGD neuropathology.[8,40] We analyzed *Gba1*$^{(flox/flox)}$; *Nestin-Cre* and *Gba1*$^{(flox/flox)}$ mice using proteomic sequencing and performed GO enrichment analysis (Figure 1). The results showed that, in addition to severe metabolic and immune disorders, *nGD* mice also exhibited cellular connectivity and synaptic dysfunction, which, once established, cannot be corrected solely by the restoration of GCase activity.[5,6] In neurons with *GBA1* mutations, GCase expressed by GBA1 is inactivated, resulting in non-degradation of the substrate, accumulation of glucose-ceramide in the neurons, sending of signals by the neurons to the surrounding microglia, activation of stationary microglia, and triggering of a neuroinflammatory cascade reaction by activated microglia.[57] Sustained glial activation leads to chronic inflammation, which in turn leads to neuronal cell death.[7] The loss of neuronal cells is usually preceded by damage to synapses and axons.[58,59]

Synaptic degeneration appears to be a hallmark of neurodegenerative diseases (NDDs).[16] In Alzheimer's disease (AD), PD and Huntington's disease (HD) synaptic failure and loss occur before neuronal loss.[60–63] *In vitro* results showed that GDNF promotes synaptogenesis in neuronal cells under the action of inhibitors and reduces neuronal death under the action of neurotoxic inducers (Figure 2). Therefore, GDNF is suitable for treating neuromuscular diseases.[33] Furthermore, AAV2-GDNF has entered clinical trials for treating PD (Clinicaltrials: NCT04167540), indicating that GDNF is a promising molecule for treating nGD neurodegeneration.[32] MRI results showed that GDNF prevented brain atrophy in *nGD* mice, indicating that

GDNF plays an important role in brain development (Figure 3). In addition, we observed morphological recovery of cortical and dopaminergic neurons following AAV9-GDNF and AAV9-GBA1-GDNF treatment. In addition, AAV9-GBA1-GDNF promotes the secretion of other growth factors that work together to restore neurodevelopmental disorders associated with nGD (Figure 5). Future studies should explore the mechanisms by which GDNF promotes the expression of other neurotrophic factors.

Generally, mature CNS neurons are highly resistant to reactive oxygen species, excitotoxicity, synaptic dysfunction, impaired protein degradation, endoplasmic reticulum stress, mitochondrial dysfunction, and inflammation.[7,64] Therefore, the normal resistance state is impaired, leading to neuronal death. Our *in vivo* and *in vitro* results showed that GDNF plays a significant role in the inhibition of neural cell apoptosis in *nGD* mice. We hypothesized that its secretion would activate downstream signaling pathways. KEGG pathway analysis of proteomic sequencing data showed that GDNF could restore autophagic lysosomal function by affecting the mTOR and PI3K-AKT pathways, which is consistent with previous studies (Figure 4).[65–68] GSK-3β/pGSK-3β is located downstream of the PI3K/AKT signaling pathway, affecting a variety of biological activities, such as cell proliferation, growth, and cell survival.[69] Accumulating evidence indicates that oxidative stress increases the expression of GSK-3β, which plays a key role in tau hyperphosphorylation and formation of nerve fiber tangles in AD brain neurons.[70,71] Combined with the therapeutic effects and proteomic analysis, it was found that GBA1 and GDNF had an additive protective effect on nGD neurons by activating the AKT/GSK3β pathway (Figure 7).

Behavioral indicators are important for pharmacodynamic evaluations in preclinical animal models.[5] After treatment with AAV9-GBA1-GDNF, the survival period of *nGD* mice was prolonged and motor coordination was restored. In a pre-experimental phase to explore the efficacy of GDNF, we attempted bilateral parenchymal administration of AAV9-GDNF, but the results showed that *nGD* mice could not survive for more than 1 month (data not shown). As previously reported, GBA1 is an important factor in restoring mouse survival and performance.[72] In *nGD* mouse models, *GBA1* mutations inactivate enzyme function, leading to the metabolite aggregation of abnormal proteins in the brain.[73] The autologous lysosome-related index, LAMP1 expression was normal in the AAV9-GBA1-GDNF treatment group, indicating that lysosomal function was restored (Figure S5). Glycolipid substrate accumulation, ceramide alterations, and other secondary lipid changes occur in diseases associated with *GBA1* mutations. This accumulation promotes neuroinflammation.[74] Additionally, an analysis of 25 clinical studies demonstrated that inflammatory cytokines NLRP3, Caspase-1, IL-1β, and other proteins in plasma were considerably elevated in blood and cerebrospinal fluid in patients with PD.[75] In this study, AAV9-GBA1-GDNF effectively suppressed neuroinflammation (Figure S4). Further studies concerning the therapeutic effects of AAV9-GBA1-GDNF on different brain regions revealed that different areas experience a certain degree of therapeutic effects (Figure S7).

In conclusion, for complex NDDs, the window for therapeutic interventions based on synaptic repair and regeneration is longer than that for toxin-clearance approaches, and such interventions can be applied at a relatively late stage of the disease to slow disease progression.[16,76] For nGD, the combination of restoration of GCase expression and augmentation of neuroprotective pathways is innovative and clinically practical. Double-target AAV9-GBA1-GDNF reversed the function of GBA1 and neuronal degeneration with no evidence of cerebral inflammation. Intraparenchymal administration of AAV9-GBA1-GDNF may provide a novel strategy and a proof-of-concept demonstration for the development of potential therapies for nGD and GBA1-related PD.

MATERIALS AND METHODS

Cells and plasmids

SH-SY5Y cells were purchased from Procell Life Science & Technology (Wuhan, China), cultured in Dulbecco's modified Eagle's medium (DMEM)/F-12 (Gibco, USA) supplemented with 10% (v/v) heat-inactivated fetal bovine serum (FBS) (DearyTech, Saibaiao, China). HT22 cells were purchased from Jiniou (Guangzhou, China). The culture medium contained DMEM (Gibco, USA) and 10% (v/v) heat-inactivated FBS. All the cell lines were cultured in an incubator at 37°C and 5% CO_2. The AAV9 rep/cap, transgene, and pAD helper plasmids for recombinant AAV9 vector production were obtained from Addgene (Cambridge, MA, USA). The transgene plasmid pAAV2/chicken β-actin (CBA)-GFP was generated using BsrgI and BglII (New England Biolabs, USA). Screened human *GBA1* (GenBank: BC003356.1) and human *GDNF* (GenBank: NM_000514.4) cDNAs were synthesized by GENEWIZ (Suzhou, China).

AAV9 viral vector production

Five types of viruses were identified. AAV9 full particles expressing GFP, GBA1, GDNF, or GBA1 linked with GDNF driven by the CBA promoter were produced using a triple transfection in HEK293 cells as previously described.[77] HEK293 cells were collected and lysed 72 h post-transfection.[78] The supernatant was then subjected to a cesium chloride gradient ultracentrifugation.[77,79] We purified rAAV2/9-*CBA-KOZAK-hGBA1-polyA* (AAV9-GBA1 1e13 vg/mL), rAAV2/9-*CBA-KOZAK-hGBA1-T2A-GDNF-polyA* (AAV9-GBA1-GDNF 1e13 vg/mL), rAAV2/9-*CBA-KOZAK-hGDNF-polyA* (AAV9-GDNF 1e13 vg/mL), rAAV2/9-*CBA-KOZAK-polyA* (AAV9-NC 1e13 vg/mL), and rAAV2/9-*CBA-KOZAK-GFP-polyA* (AAV9-GFP 1e13 vg/mL).

Animals

Ethics statement

The animal procedures and welfare were approved by the China Medical University Animal Welfare and Ethical Review Board (CMU 2022082).

Generation of Gba1 *knockout mice*

The *Gba1* conditional knockout mice, *Gba1*$^{(flox/flox)}$; *Nestin-Cre*, were generated by CRISPR technology according to the previous study.[40] *Gba1*$^{(flox/flox)}$ and *Gba1*$^{(flox/+)}$;*Nestin-Cre* mice were purchased from

Cyagen Biosciences Inc. (Jiangsu, China). *Gba1*$^{(flox/flox)}$ mice was crossed with *Gba1*$^{(flox/+)}$*; Nestin-Cre* mice to generate *Gba1*$^{(flox/flox)}$*; Nestin-Cre*, which conditionally knocked *Gba1* in neuronal precursors. After birth, the mice were genotyped, we selected *Gba1*$^{(flox/flox)}$*; Nestin-Cre (nGD)* for treatment.

Experimental design

At postnatal day 1 (P1), all the mice were divided into six groups: the *Gba1*$^{(flox/flox)}$ mice with double-point phosphate-buffered saline (PBS) group (2 μL/point), the *nGD* mice with double-point PBS group (2 μL/point), the *nGD* mice with single-point AAV9-GBA1 (2 μL/point), the *nGD* mice with double-point AAV9-GBA1 (2 μL/point), the *nGD* mice with different drug combinations of AAV9-GBA1 on one side of the brain and AAV9-GDNF on the opposite side (2 μL/point), and the *nGD* mice with double-point AAV9-GBA1-GDNF (2 μL/point). The number of mice in each group was greater than or equal to eight. Single-point means injection into one cerebral hemisphere, double-point means inject into both cerebral hemispheres, respectively.

The *Gba1*$^{(flox/flox)}$ mice group was set as the control. The double-point PBS group was set as the disease group. The single-point AAV9-GBA1 group was designed to be compared with the double-point AAV9-GBA1 for a dose-dependent study. Besides, the different drug combinations of AAV9-GBA1 on one side of the brain and AAV9-GDNF on the opposite side was designed to compare the effects of GBA1 and GDNF on disease phenotypes in the same individual. To compare the effects of GBA1 and GDNF on *nGD* phenotypes, the double-point AAV9-GBA1 and the double-point AAV9-GBA1-GDNF were designed (n = 8 per group).

Pups were anesthetized on ice for 30 s and injected at P1. For intraparenchymal delivery, a Hamilton syringe with a 33-G needle and a measuring range of 2.5 μL was placed at the distal one-third of the Lambda line. The needle was inserted vertically, with a depth of 2 mm and an injection speed of 2 min/point. After complete administration of the drug, the syringe was left in place for 1 min and then the needle was quickly withdrawn to avoid bleeding. Fully recovered pups were returned to their cages. The mice were observed and weighed twice weekly after administration. The dose of the injection site of the *Gba1*$^{(flox/flox)}$ mice used in the safety evaluation was consistent with that in the *nGD* mice.

Culture of primary hippocampal neurons

After genotype sorting, newborn (P1) *Gba1*$^{(flox/flox)}$ and *nGD* mice were obtained from SPF Laboratory Animal Center, China Medical University. The hippocampal neurons were isolated, as described previously.[80] After sterilizing the mouse skin with 75% alcohol, intact brain tissue was removed in HBSS buffer. The vascular membrane was removed and hippocampal tissue was isolated under an optical microscope. After trypsin digestion, single-cell suspension was prepared by DMEM/F-12 with 10% FBS,1% P/S (v/v) medium. Neurobasal with B27 (Gibco, USA) as the complete medium was changed 2 h after inoculation, then AAV9-GBA1 or AAV9-GBA1-GDNF (1e10vg) was added. Protein expression was evaluated after 48 h of treatment.

Western blotting

Tissue samples were collected in phosphate-buffered saline (PBS) on ice, frozen, ground evenly in liquid nitrogen, and dissolved in radioimmunoprecipitation assay (RIPA) buffer (Solarbio Institute of Biotechnology, Beijing, China). Proteins were quantified using a bicinchoninic acid (BCA) protein assay kit (Solarbio Institute of Biotechnology). Equal amounts of protein samples (20–60 μg) were separated using 10%–12% sodium dodecyl sulfate-polyacrylamide gel electrophoresis and electrophoretically transferred onto polyvinylidene fluoride membranes. To avoid non-specific binding, membranes were blocked with 5% nonfat milk for 45 min at room temperature, and the phosphorylation index was blocked with 5% bovine serum albumin (BSA) at 4°C overnight. Subsequently, the membranes were incubated with antibody for 2 h at room temperature or 4°C overnight. After washing, the membranes were incubated with horseradish peroxidase-conjugated secondary antibodies for 1 h at room temperature. After washing three times with Tris-buffered saline containing 1% Tween 20, the immunoblots were visualized using an enhanced chemiluminescence kit (PN 180–5001, Tanon, Shanghai, China) and scanned using Tanon AllDoc_x. The integrated density values were calculated using ImageJ 9.0. The antibody information is described in Table S1.

HT22 was treated with 5 μM Laduviglusib (MedChemExpress, China) and 6 h later was treated with AAV9-GBA1-GDNF. Then 48 h later HT22 samples were collected in RIPA on ice, followed by ultrasonic lysis. As with tissue, protein quantification and western blotting were performed.

Real-time PCR assay

Total RNAs were extracted from the brain tissue using TRIzol reagent (PN DP424, TIANGEN, Beijing, China). The concentration and quality of the RNA was subsequently determined using a Nano Photometer N50 Touch (Implen, Germany). TakaRa Prime Script RT master mix (RR036A) was used to reverse transcribe total RNA (500 ng) into cDNA. qPCR was performed on a Light Cycler 96 real-time system (Roche, Switzerland) using a pair of primer-specific genes. GAPDH was used as an internal reference. The relative expression values were calculated using relative quantification ($2^{-\Delta\Delta Ct}$). The gene and primer information are described in Table S2.

Flow cytometry

The spleen was filtered through a 70-μm cell strainer (Falcon, USA) to obtain a single-cell suspension. The cells were subsequently subjected to lysis of the red blood cells using 2 mL red blood cell lysis buffer (Absin, China). Cell suspensions were stained using the Fixable Viability Kit (BioLegend, USA) for 20 min to eliminate dead cells, and subsequently incubated for 15 min with a fragment crystallizable block antibody. Cell suspensions were then stained with the corresponding FITC anti-mouse CD4 antibody, PerCP/Cy5.5 anti-mouse CD8 antibody, PE anti-mouse CD45 antibody, and PB anti-mouse

CD49 antibody (BioLegend) for 30 min at 4°C. The stained cells were analyzed using a BD Fortessa (BD Biosciences, USA). Flow cytometry data were analyzed using Flow Jo V10.

Immunofluorescence assay

The SH-SY5Y cells or hippocampal neurons were seeded in a 24-well plate on a circular slide (PN 801010; NEST, USA). AAV9-BDNF, AAV9-CDNF, AAV9-GDNF, and AAV9-NRTN were subsequently added (1e9/well) and then treated with Conduritol B-epoxide (CBE) (Sigma-Aldrich, Germany). The cells were captured after 48 h under the white light of a microscope and washed three times with PBS buffer. Cells were then fixed with 4% paraformaldehyde for 20 min and permeabilized with 0.5% Triton X-100 for 40 min at room temperature. After blocking with 5% BSA for 30 min, the cells were incubated with MAP2 primary antibodies (1:100 dilution, PN ab32127, Abcam) at 4°C overnight. The cells were incubated with DyLight 488-conjugated goat anti-mouse (1:1,000 dilution, PN ab196379, Abcam) for 1 h at room temperature. The stained cells were visualized under a confocal microscope (Nikon AX).

Cell proliferation

SH-SY5Y and HT22 cells were seeded in 96-well plates. After a suitable drug concentration was determined, AAV9-GDNF (1e9vg/well) was added, followed by treatment with 1.5 mM, 2.25 mM, or 3 mM 1-methyl-4-phenyl-1,2,3,6-tetrahydropyridine (MPTP) (Sigma-Aldrich, Germany). Cells were washed with PBS, and fresh medium was replaced at 48 h after infection with the virus. Then CCK8 detection reagent (MedChemExpress, China) was added; after 2 h the absorption intensity was measured at 450 nm.

GCase activity assay

GCase activity was determined with the established protocol using a synthetic substrate, 4-methylumbelliferone-β-glucopyranoside, as previously described.[81] Frozen brain samples were homogenized with distilled water on ice, and the total protein concentration was measured using the BCA assay. The samples were subsequently added at 10 μg/well into a 96-well enzyme label plate. Samples were incubated with the substrate for 2 h at 37°C. The reaction was stopped using 1 M glycine buffer at pH 10.4. The fluorescence of the standard (1 nM of 4-methylumbelliferone) and the samples was measured (BioTek Cytation5, USA). The excitation and emission wave lengths were 360 nm and 490 nm, respectively.

Immunohistochemistry assay

Three to 4 months after injection, mice were culled by terminal transcardial perfusion using PBS. Brains were harvested following euthanasia using isoflurane, cardiac puncture, and perfusion of 1% paraformaldehyde, as previously described.[81] After that, the mice brains were fixed in 4% paraformaldehyde. The largest portion of the hippocampus was cut using a tissue membrane to a thickness of 40 μm. After antigen recovery, slides were blocked with 3% BSA, washed with PBS, and incubated with primary antibodies overnight (Table S1). The slides were subsequently incubated with a secondary antibody and counterstained with 4′,6-diamidino-2-phenylindole for 10 min (KeyGEN BioTECH PN KGA215, China). Finally, the slides were dehydrated. Images were acquired using a Nikon DS-U3 controller equipped with a Nikon microscope E100.

Proteomic sequencing analysis

At 3 months post-intraparenchymal delivery, brains from the mice of the *nGD*-PBS (P21), *Gba1*$^{(flox/flox)}$-PBS, *nGD*-AAV9-GBA1, and *nGD*-AAV9-GBA1-GDNF groups were extracted and frozen in liquid nitrogen. Proteins were identified via BCA quantification and Coomassie brilliant blue staining. Subsequently, peptide enzymatic hydrolysis, LC-MS/MS, and library-building analyses were conducted. We generated a Venn plot using a box based on the number of DEPs (Sangerbox: http://vip.sangerbox.com/home.html).

GO annotation

The protein sequences of the selected DEPs were locally searched using the NCBI BLAST+ client software (ncbi-blast-2.2.28+-win32.exe) and Inter Pro Scan to find homologous sequences. The GO terms were subsequently mapped, and the sequences were annotated using Blast2GO. The GO annotation results were plotted using the R Programming Language 3.0.1.

KEGG annotation

Following annotation, the studied proteins were blasted against the online KEGG database (KEGG database: http://geneontology.org/) to retrieve their KEGG orthology identifications and subsequently mapped to KEGG pathways.

Behavioral assessment

Rota rod test

Mice were trained for 3 days before performing the tests; *nGD* P21 was set as the minimum value. The rotarod (Ugo Basil-47650, Italy) was set to accelerate from 10 rpm to 40 rpm in 10 s, and the time at which the mice fell off the rod was recorded. The endpoint of the experiment was set at 300 s.

Strain relief test

In the pull test, the limbs of the mice were placed on a barbed wire, and the force the mouse exerted on the barbed wire when it pulled back produced a reading that was recorded (Ugo Basil-47200, Italy). *nGD* P21 was set as the minimum value.

Balance beam test

Mice were trained for 3 days before performing the tests, *nGD* P21 was set as the maximum value. The balance beam experiment involved setting up a 1-m-long stick at a height and setting the starting and stopping positions of the mice at both ends of the stick. The time required for the mice to pass through the balance beam was recorded.

MRI

Mice were placed in a respiratory anesthesia machine with isoflurane for 5 min. Mice were placed in a photographic chamber with a heating pad and a heart rate monitor (BioSpec 3T, Bruker) when they lost

limb sensation. The first step was to correct the head positions of the mice. Subsequently, T1 and T2 images were run. The mice were placed back into the cage after they woke up.

Statistical analyses

Statistical analysis was performed using GraphPad Prism 9.0 software. One-way ANOVA was used for univariate analysis. $p < 0.05$ was considered statistically significant.

DATA AVAILABILITY

The data used or analyzed during the current study are available from the corresponding author on reasonable request.

ACKNOWLEDGMENTS

We appreciate the Research and Experiment Center of China Medical University for providing the instruments and equipment of the public experimental platform. This work is supported by the National Natural Science Foundation of China (No. 82070826).

AUTHOR CONTRIBUTIONS

Conceptualization, Visualization, Project administration – Y.M., J.Z., R.F., Y.L., and G.L.; Investigation – Y.M., J.Z., Y.L., and G.L.; Methodology, Data curation, and Formal Analysis – Y.M., J.Z., W.P., W.Z., Q.G.; Resources and Software – Q.G., X.H., Y.L., and G.L.; Writing – original draft – Y.M., R.F., Y.L., and G.L.; Writing – review & editing – Y.M., R.F., Y.L., and G.L.; Supervision – Y.L. and G.L.; Funding acquisition – Y.L. and G.L.

DECLARATION OF INTERESTS

The authors declare no competing interests.

SUPPLEMENTAL INFORMATION

Supplemental information can be found online at https://doi.org/10.1016/j.omtn.2025.102506.

REFERENCES

1. Schiffmann, R., Sevigny, J., Rolfs, A., Davies, E.H., Goker-Alpan, O., Abdelwahab, M., Vellodi, A., Mengel, E., Lukina, E., Yoo, H.W., et al. (2020). The definition of neuronopathic Gaucher disease. J. Inherit. Metab. Dis. *43*, 1056–1059.
2. Biegstraaten, M., van Schaik, I.N., Aerts, J.M.F.G., Hollak, C.E.M., and Hollak, C.E. (2008). 'Non-neuronopathic' Gaucher disease reconsidered. Prevalence of neurological manifestations in a Dutch cohort of type I Gaucher disease patients and a systematic review of the literature. J. Inherit. Metab. Dis. *31*, 337–349.
3. Daykin, E.C., Ryan, E., and Sidransky, E. (2021). Diagnosing neuronopathic Gaucher disease: New considerations and challenges in assigning Gaucher phenotypes. Mol. Genet. Metabol. *132*, 49–58.
4. Weiss, K., Gonzalez, A., Lopez, G., Pedoeim, L., Groden, C., and Sidransky, E. (2015). The clinical management of Type 2 Gaucher disease. Mol. Genet. Metabol. *114*, 110–122.
5. Arévalo, N.B., Lamaizon, C.M., Cavieres, V.A., Burgos, P.V., Álvarez, A.R., Yañez, M.J., and Zanlungo, S. (2022). Neuronopathic Gaucher disease: Beyond lysosomal dysfunction. Front. Mol. Neurosci. *15*, 934820.
6. Boddupalli, C.S., Nair, S., Belinsky, G., Gans, J., Teeple, E., Nguyen, T.H., Mehta, S., Guo, L., Kramer, M.L., Ruan, J., et al. (2022). Neuroinflammation in neuronopathic Gaucher disease: Role of microglia and NK cells, biomarkers, and response to substrate reduction therapy. Elife *11*, e79830.
7. Vitner, E.B., Farfel-Becker, T., Eilam, R., Biton, I., and Futerman, A.H. (2012). Contribution of brain inflammation to neuronal cell death in neuronopathic forms of Gaucher's disease. Brain *135*, 1724–1735.
8. Farfel-Becker, T., Vitner, E.B., Pressey, S.N.R., Eilam, R., Cooper, J.D., and Futerman, A.H. (2011). Spatial and temporal correlation between neuron loss and neuroinflammation in a mouse model of neuronopathic Gaucher disease. Hum. Mol. Genet. *20*, 1375–1386.
9. Goker-Alpan, O., Schiffmann, R., Park, J.K., Stubblefield, B.K., Tayebi, N., and Sidransky, E. (2003). Phenotypic continuum in neuronopathic Gaucher disease: an intermediate phenotype between type 2 and type 3. J. Pediatr. *143*, 273–276.
10. García-Sanz, P., Orgaz, L., Fuentes, J.M., Vicario, C., and Moratalla, R. (2018). Cholesterol and multilamellar bodies: Lysosomal dysfunction in GBA-Parkinson disease. Autophagy *14*, 717–718.
11. Riboldi, G.M., and Di Fonzo, A.B. (2019). GBA, Gaucher Disease, and Parkinson's Disease: From Genetic to Clinic to New Therapeutic Approaches. Cells *8*, 364.
12. Stirnemann, J., Belmatoug, N., Camou, F., Serratrice, C., Froissart, R., Caillaud, C., Levade, T., Astudillo, L., Serratrice, J., Brassier, A., et al. (2017). A Review of Gaucher Disease Pathophysiology, Clinical Presentation and Treatments. Int. J. Mol. Sci. *18*, 441.
13. Kaye, E.M., Ullman, M.D., Wilson, E.R., and Barranger, J.A. (1986). Type 2 and type 3 Gaucher disease: a morphological and biochemical study. Ann. Neurol. *20*, 223–230.
14. Andreone, B.J., Larhammar, M., and Lewcock, J.W. (2020). Cell Death and Neurodegeneration. Cold Spring Harbor Perspect. Biol. *12*, a036434.
15. Grabowski, G.A., Antommaria, A.H.M., Kolodny, E.H., and Mistry, P.K. (2021). Gaucher disease: Basic and translational science needs for more complete therapy and management. Mol. Genet. Metabol. *132*, 59–75.
16. Wilson, D.M., 3rd, Cookson, M.R., Van Den Bosch, L., Zetterberg, H., Holtzman, D.M., and Dewachter, I. (2023). Hallmarks of neurodegenerative diseases. Cell *186*, 693–714.
17. Grabowski, G.A. (2008). Phenotype, diagnosis, and treatment of Gaucher's disease. Lancet *372*, 1263–1271.
18. Smith, L., and Schapira, A.H.V. (2022). GBA Variants and Parkinson Disease: Mechanisms and Treatments. Cells *11*, 1261.
19. Gupta, N., Oppenheim, I.M., Kauvar, E.F., Tayebi, N., and Sidransky, E. (2011). Type 2 Gaucher disease: phenotypic variation and genotypic heterogeneity. Blood Cells Mol. Dis. *46*, 75–84.
20. Mistry, P.K., Lopez, G., Schiffmann, R., Barton, N.W., Weinreb, N.J., and Sidransky, E. (2017). Gaucher disease: Progress and ongoing challenges. Mol. Genet. Metabol. *120*, 8–21.
21. Wyatt, K., Henley, W., Anderson, L., Anderson, R., Nikolaou, V., Stein, K., Klinger, L., Hughes, D., Waldek, S., Lachmann, R., et al. (2012). The effectiveness and cost-effectiveness of enzyme and substrate replacement therapies: a longitudinal cohort study of people with lysosomal storage disorders. Health Technol. Assess. *16*, 1–543.
22. Sawamoto, K., Chen, H.H., Alméciga-Díaz, C.J., Mason, R.W., and Tomatsu, S. (2018). Gene therapy for Mucopolysaccharidoses. Mol. Genet. Metabol. *123*, 59–68.
23. Desnick, R.J., and Schuchman, E.H. (2002). Enzyme replacement and enhancement therapies: lessons from lysosomal disorders. Nat. Rev. Genet. *3*, 954–966.
24. Migita, M., Hamada, H., Fujimura, J., Watanabe, A., Shimada, T., and Fukunaga, Y. (2003). Glucocerebrosidase level in the cerebrospinal fluid during enzyme replacement therapy–unsuccessful treatment of the neurological abnormality in type 2 Gaucher disease. Eur. J. Pediatr. *162*, 524–525.
25. Vellodi, A., Bembi, B., de Villemeur, T.B., Collin-Histed, T., Erikson, A., Mengel, E., Rolfs, A., and Tylki-Szymanska, A.; Neuronopathic Gaucher Disease Task Force of the European Working Group on Gaucher Disease (2001). Management of neuronopathic Gaucher disease: a European consensus. J. Inherit. Metab. Dis. *24*, 319–327.
26. Tajima, A., Yokoi, T., Ariga, M., Ito, T., Kaneshiro, E., Eto, Y., and Ida, H. (2009). Clinical and genetic study of Japanese patients with type 3 Gaucher disease. Mol. Genet. Metabol. *97*, 272–277.
27. Kim, J., He, M.J., Widmann, A.K., and Lee, F.S. (2024). The role of neurotrophic factors in novel, rapid psychiatric treatments. Neuropsychopharmacology *49*, 227–245.
28. Khazaei, M., Ahuja, C.S., Nakashima, H., Nagoshi, N., Li, L., Wang, J., Chio, J., Badner, A., Seligman, D., Ichise, A., et al. (2020). GDNF rescues the fate of neural progenitor grafts by attenuating Notch signals in the injured spinal cord in rodents. Sci. Transl. Med. *12*, eaau3538.
29. De Lorenzo, F., Lüningschrör, P., Nam, J., Beckett, L., Pilotto, F., Galli, E., Lindholm, P., Rüdt von Collenberg, C., Mungwa, S.T., Jablonka, S., et al. (2023). CDNF rescues motor neurons in models of amyotrophic lateral sclerosis by targeting endoplasmic reticulum stress. Brain *146*, 3783–3799.

30. Espinoza, S., Scarpato, M., Damiani, D., Managò, F., Mereu, M., Contestabile, A., Peruzzo, O., Carninci, P., Santoro, C., Papaleo, F., et al. (2020). SINEUP Non-coding RNA Targeting GDNF Rescues Motor Deficits and Neurodegeneration in a Mouse Model of Parkinson's Disease. Mol. Ther. *28*, 642–652.

31. Gash, D.M., Gerhardt, G.A., Bradley, L.H., Wagner, R., and Slevin, J.T. (2020). GDNF clinical trials for Parkinson's disease: a critical human dimension. Cell Tissue Res. *382*, 65–70.

32. Barker, R.A., Björklund, A., Gash, D.M., Whone, A., Van Laar, A., Kordower, J.H., Bankiewicz, K., Kieburtz, K., Saarma, M., Booms, S., et al. (2020). GDNF and Parkinson's Disease: Where Next? A Summary from a Recent Workshop. J. Parkinsons Dis. *10*, 875–891.

33. Cintrón-Colón, A.F., Almeida-Alves, G., Boynton, A.M., and Spitsbergen, J.M. (2020). GDNF synthesis, signaling, and retrograde transport in motor neurons. Cell Tissue Res. *382*, 47–56.

34. Mendell, J.R., Al-Zaidy, S.A., Rodino-Klapac, L.R., Goodspeed, K., Gray, S.J., Kay, C.N., Boye, S.L., Boye, S.E., George, L.A., Salabarria, S., et al. (2021). Current Clinical Applications of In Vivo Gene Therapy with AAVs. Mol. Ther. *29*, 464–488.

35. Wang, D., Tai, P.W.L., and Gao, G. (2019). Adeno-associated virus vector as a platform for gene therapy delivery. Nat. Rev. Drug Discov. *18*, 358–378.

36. Tosolini, A.P., and Sleigh, J.N. (2017). Motor Neuron Gene Therapy: Lessons from Spinal Muscular Atrophy for Amyotrophic Lateral Sclerosis. Front. Mol. Neurosci. *10*, 405.

37. Amado, D.A., and Davidson, B.L. (2021). Gene therapy for ALS: A review. Mol. Ther. *29*, 3345–3358.

38. Cappella, M., Pradat, P.F., Querin, G., and Biferi, M.G. (2021). Beyond the Traditional Clinical Trials for Amyotrophic Lateral Sclerosis and The Future Impact of Gene Therapy. J. Neuromuscul. Dis. *8*, 25–38.

39. Chen, S.K., Hawley, Z.C.E., Zavodszky, M.I., Hana, S., Ferretti, D., Grubor, B., Hawes, M., Xu, S., Hamann, S., Marsh, G., et al. (2023). Efficacy and safety of a SOD1-targeting artificial miRNA delivered by AAV9 in mice are impacted by miRNA scaffold selection. Mol. Ther. Nucleic Acids *34*, 102057.

40. Enquist, I.B., Lo Bianco, C., Ooka, A., Nilsson, E., Månsson, J.E., Ehinger, M., Richter, J., Brady, R.O., Kirik, D., and Karlsson, S. (2007). Murine models of acute neuronopathic Gaucher disease. Proc. Natl. Acad. Sci. USA *104*, 17483–17488.

41. Vardi, A., Zigdon, H., Meshcheriakova, A., Klein, A.D., Yaacobi, C., Eilam, R., Kenwood, B.M., Rahim, A.A., Massaro, G., Merrill, A.H., Jr., et al. (2016). Delineating pathological pathways in a chemically induced mouse model of Gaucher disease. J. Pathol. *239*, 496–509.

42. Bondar, C., Ormazabal, M., Crivaro, A., Ferreyra-Compagnucci, M., Delpino, M.V., Rozenfeld, P.A., and Mucci, J.M. (2017). Osteocyte Alterations Induce Osteoclastogenesis in an In Vitro Model of Gaucher Disease. Int. J. Mol. Sci. *18*, 112.

43. Molofsky, A.V., Krencik, R., Ullian, E.M., Tsai, H.H., Deneen, B., Richardson, W.D., Barres, B.A., and Rowitch, D.H. (2012). Astrocytes and disease: a neurodevelopmental perspective. Genes Dev. *26*, 891–907.

44. Lee, J., Hyeon, S.J., Im, H., Ryu, H., Kim, Y., and Ryu, H. (2016). Astrocytes and Microglia as Non-cell Autonomous Players in the Pathogenesis of ALS. Exp. Neurobiol. *25*, 233–240.

45. MacMahon Copas, A.N., McComish, S.F., Fletcher, J.M., and Caldwell, M.A. (2021). The Pathogenesis of Parkinson's Disease: A Complex Interplay Between Astrocytes, Microglia, and T Lymphocytes? Front. Neurol. *12*, 666737.

46. Schiffmann, R., Fitzgibbon, E.J., Harris, C., DeVile, C., Davies, E.H., Abel, L., van Schaik, I.N., Benko, W., Timmons, M., Ries, M., and Vellodi, A. (2008). Randomized, controlled trial of miglustat in Gaucher's disease type 3. Ann. Neurol. *64*, 514–522.

47. Sako, S., Oishi, K., Ida, H., and Imagawa, E. (2024). Allele frequency of pathogenic variants causing acid sphingomyelinase deficiency and Gaucher disease in the general Japanese population. Hum. Genome Var. *11*, 24.

48. Nalysnyk, L., Rotella, P., Simeone, J.C., Hamed, A., and Weinreb, N. (2017). Gaucher disease epidemiology and natural history: a comprehensive review of the literature. Hematology *22*, 65–73.

49. Shemesh, E., Deroma, L., Bembi, B., Deegan, P., Hollak, C., Weinreb, N.J., and Cox, T.M. (2015). Enzyme replacement and substrate reduction therapy for Gaucher disease. Cochrane Database Syst. Rev. *2015*, Cd010324.

50. De, B.P., Rosenberg, J.B., Selvan, N., Wilson, I., Yusufzai, N., Greco, A., Kaminsky, S.M., Heier, L.A., Ricart Arbona, R.J., Miranda, I.C., et al. (2023). Assessment of Safety and Biodistribution of AAVrh.10hCLN2 Following Intracisternal Administration in Nonhuman Primates for the Treatment of CLN2 Batten Disease. Hum. Gene Ther. *34*, 905–916.

51. Yang, K., Li, T., Geng, Y., Zhang, R., Xu, Z., Wu, J., Yuan, Y., Zhang, Y., Qiu, Z., and Li, F. (2024). Protocol for the neonatal intracerebroventricular delivery of adeno-associated viral vectors for brain restoration of MECP2 for Rett syndrome. STAR Protoc. *5*, 103344.

52. Meseck, E.K., Guibinga, G., Wang, S., McElroy, C., Hudry, E., and Mansfield, K. (2022). Intrathecal sc-AAV9-CB-GFP: Systemic Distribution Predominates Following Single-Dose Administration in Cynomolgus Macaques. Toxicol. Pathol. *50*, 415–431.

53. Hinderer, C., Katz, N., Buza, E.L., Dyer, C., Goode, T., Bell, P., Richman, L.K., and Wilson, J.M. (2018). Severe Toxicity in Nonhuman Primates and Piglets Following High-Dose Intravenous Administration of an Adeno-Associated Virus Vector Expressing Human SMN. Hum. Gene Ther. *29*, 285–298.

54. Gray, S.J., Nagabhushan Kalburgi, S., McCown, T.J., and Jude Samulski, R. (2013). Global CNS gene delivery and evasion of anti-AAV-neutralizing antibodies by intrathecal AAV administration in non-human primates. Gene Ther. *20*, 450–459.

55. Chen, W., Hu, Y., and Ju, D. (2020). Gene therapy for neurodegenerative disorders: advances, insights and prospects. Acta Pharm. Sin. B *10*, 1347–1359.

56. Kang, L., Jin, S., Wang, J., Lv, Z., Xin, C., Tan, C., Zhao, M., Wang, L., and Liu, J. (2023). AAV vectors applied to the treatment of CNS disorders: Clinical status and challenges. J. Contr. Release *355*, 458–473.

57. Shimizu, T., Schutt, C.R., Izumi, Y., Tomiyasu, N., Omahdi, Z., Kano, K., Takamatsu, H., Aoki, J., Bamba, T., Kumanogoh, A., et al. (2023). Direct activation of microglia by β-glucosylceramide causes phagocytosis of neurons that exacerbates Gaucher disease. Immunity *56*, 307–319.e8.

58. Jamjoom, A.A.B., Rhodes, J., Andrews, P.J.D., and Grant, S.G.N. (2021). The synapse in traumatic brain injury. Brain *144*, 18–31.

59. Tagliaferro, P., and Burke, R.E. (2016). Retrograde Axonal Degeneration in Parkinson Disease. J. Parkinsons Dis. *6*, 1–15.

60. Selkoe, D.J. (2002). Alzheimer's disease is a synaptic failure. Science *298*, 789–791.

61. Soukup, S.F., Vanhauwaert, R., and Verstreken, P. (2018). Parkinson's disease: convergence on synaptic homeostasis. EMBO J. *37*, e98960.

62. Cheng, H.C., Ulane, C.M., and Burke, R.E. (2010). Clinical progression in Parkinson disease and the neurobiology of axons. Ann. Neurol. *67*, 715–725.

63. Li, J.Y., Plomann, M., and Brundin, P. (2003). Huntington's disease: a synaptopathy? Trends Mol. Med. *9*, 414–420.

64. Chi, H., Chang, H.Y., and Sang, T.K. (2018). Neuronal Cell Death Mechanisms in Major Neurodegenerative Diseases. Int. J. Mol. Sci. *19*, 3082.

65. Chmielarz, P., Er, Ş., Konovalova, J., Bandres, L., Hlushchuk, I., Albert, K., Panhelainen, A., Luk, K., Airavaara, M., and Domanskyi, A. (2020). GDNF/RET Signaling Pathway Activation Eliminates Lewy Body Pathology in Midbrain Dopamine Neurons. Mov. Disord. *35*, 2279–2289.

66. Goyal, A., Agrawal, A., Verma, A., and Dubey, N. (2023). The PI3K-AKT pathway: A plausible therapeutic target in Parkinson's disease. Exp. Mol. Pathol. *129*, 104846.

67. Kramer, E.R., and Liss, B. (2015). GDNF-Ret signaling in midbrain dopaminergic neurons and its implication for Parkinson disease. FEBS Lett. *589*, 3760–3772.

68. Er, S., and Airavaara, M. (2023). Protective mechanisms by glial cell line-derived neurotrophic factor and cerebral dopamine neurotrophic factor against the α-synuclein accumulation in Parkinson's disease. Biochem. Soc. Trans. *51*, 245–257.

69. Majewska, E., and Szeliga, M. (2017). AKT/GSK3β Signaling in Glioblastoma. Neurochem. Res. *42*, 918–924.

70. Dinda, B., Dinda, M., Kulsi, G., Chakraborty, A., and Dinda, S. (2019). Therapeutic potentials of plant iridoids in Alzheimer's and Parkinson's diseases: A review. Eur. J. Med. Chem. *169*, 185–199.

71. Yang, L., Wang, H., Liu, L., and Xie, A. (2018). The Role of Insulin/IGF-1/PI3K/Akt/GSK3β Signaling in Parkinson's Disease Dementia. Front. Neurosci. *12*, 73.

72. Zheng, W., and Fan, D. (2022). Glucocerebrosidase Mutations Cause Mitochondrial and Lysosomal Dysfunction in Parkinson's Disease: Pathogenesis and Therapeutic Implications. Front. Aging Neurosci. *14*, 851135.

73. Allende, M.L., Zhu, H., Kono, M., Hoachlander-Hobby, L.E., Huso, V.L., and Proia, R.L. (2021). Genetic defects in the sphingolipid degradation pathway and their effects on microglia in neurodegenerative disease. Cell. Signal. *78*, 109879.

74. Abeliovich, A., Hefti, F., and Sevigny, J. (2021). Gene Therapy for Parkinson's Disease Associated with GBA1 Mutations. J. Parkinsons Dis. *11*, S183–S188.

75. Brandebura, A.N., Paumier, A., Onur, T.S., and Allen, N.J. (2023). Astrocyte contribution to dysfunction, risk and progression in neurodegenerative disorders. Nat. Rev. Neurosci. *24*, 23–39.

76. Lu, B., Nagappan, G., Guan, X., Nathan, P.J., and Wren, P. (2013). BDNF-based synaptic repair as a disease-modifying strategy for neurodegenerative diseases. Nat. Rev. Neurosci. *14*, 401–416.

77. Meng, Y., Sun, D., Qin, Y., Dong, X., Luo, G., and Liu, Y. (2021). Cell-penetrating peptides enhance the transduction of adeno-associated virus serotype 9 in the central nervous system. Mol. Ther. Methods Clin. Dev. *21*, 28–41.

78. Ling, C., Yu, C., Wang, C., Yang, M., Yang, H., Yang, K., He, Y., Shen, Y., Tang, S., Yu, X., et al. (2024). rAAV capsid mutants eliminate leaky expression from DNA donor template for homologous recombination. Nucleic Acids Res. *52*, 6518–6531.

79. Kohlbrenner, E., and Weber, T. (2017). Production and Characterization of Vectors Based on the Cardiotropic AAV Serotype 9. Methods Mol. Biol. *1521*, 91–107.

80. Seibenhener, M.L., and Wooten, M.W. (2012). Isolation and culture of hippocampal neurons from prenatal mice. J. Vis. Exp. 3634. https://doi.org/10.3791/3634.

81. Massaro, G., Mattar, C.N.Z., Wong, A.M.S., Sirka, E., Buckley, S.M.K., Herbert, B.R., Karlsson, S., Perocheau, D.P., Burke, D., Heales, S., et al. (2018). Fetal gene therapy for neurodegenerative disease of infants. Nat. Med. *24*, 1317–1323.

OMTN, Volume *36*

Supplemental information

AAV-mediated GBA1 and GDNF rescue neurological defects in a murine model of neuronopathic Gaucher disease

Yuan Meng, Jiale Zhang, Ruoyue Fan, Wei Pang, Wanyang Zeng, Qingguo Guo, Xuefei Han, Ying Liu, and Guangzuo Luo

Table S1 The antibody used in study

Antibody name	Catalog	Application
anti-Rabbit MAP2	abcam ab32127	IF 1:100
anti-Rabbit LAMP1	Servicebio GB112949	IHC 1:500
anti-Rabbit LAMP1	abcam ab24170	WB 1:1000<br>IF 1:400
anti-Rabbit LC3B	NOVUS NB600-1384	WB 1:1000<br>IHC 1:500
anti-Rabbit α-syn(S129)	abcam ab51253	WB 1:1000
anti-Rabbit CD68	Servicebio GB113109	IHC 1:500
anti-Rabbit CD68	Immunoway YT5210	WB 1:1000
anti-Rabbit GFAP	Servicebio GB11096	WB 1:1000<br>IHC 1:500
anti-Rabbit IBAI	CST 17198T	WB 1:1000
anti-Rabbit IL6	Immunoway YT5348	WB 1:1000
anti-Rabbit IL1β	Immunoway YT5201	WB 1:1000
anti-Rabbit TNFα	Immunoway YT4689	WB 1:1000
anti-Rabbit MAP2	abcam ab5392	WB 1:1000
anti-Rabbit Nurr1	boster PB0332	WB 1:1000
anti-Rabbit TH	proteintech 25859-1-AP	WB 1:1000<br>IHC 1:500
anti-Rabbit NeuN	Servicebio GB11138	WB 1:1000
anti-Rabbit GDNF	abcam ab18956	WB 1:1000
anti-Rabbit β-actin	proteintech 20536-1-AP	WB 1:1000
anti-Rabbit Caspase 3	boster A00334-1	WB 1:1000
anti-Rabbit Bax	boster BA0315-2	WB 1:1000
anti-Rabbit Bcl-2	boster A00040-1	WB 1:1000
anti-Rabbit AKT	Cell Signaling Technology 4691	WB 1:1000
anti-Rabbit pAKT	Cell Signaling Technology 4060	WB 1:1000
anti-Rabbit pGSK3β	Cell Signaling Technology 5558T	WB 1:1000
anti-Rabbit Synapsin-1	Cell Signaling Technology 5297	WB 1:1000
anti-Rabbit α-syn	Cell Signaling Technology 4179	WB 1:1000<br>IF 1:400
anti-Rabbit Caspase 9	Cell Signaling Technology 9508T	WB 1:1000
anti-Rabbit Glucocerebrosidase	Sigma-Aldrich G4171	WB 1:1000

Antibody name	Catalog	Application
anti-Rabbit GFP	Proteintech 50430-2-AP	WB 1:5000
anti-Rabbit GAPDH	Proteintech 10494-1-AP	WB 1:5000
HRP-conjugated Goat Anti-Rabbit IgG(H+L)	Proteintech SA00001-2	WB 1:5000
CoraLite488-conjugated Goat Anti-Rabbit IgG(H+L)	Proteintech SA00013-2	IF 1:1000

Table S2 The Sequence used in RT-PCR

Gene name	Sequence (5'->3')
m*Gfap*-F	GCAGACCTCACAGACGTTGCT
m*Gfap*-R	AGGCTGGTTTCTCGGATCTGG
m*Tnfα*-F	CGTCAGCCGATTTGCTATCT
m*Tnfα*-R	CGGACTCCGCAAAGTCTAAG
m*Inos*-F	CCTCCTTTGCCTCTCACTCTTC
m*Inos*-R	AGTATTAGAGCGGTGGCATGGT
m*Il6*-F	ATGGATGCTACCAAACTGGAT
m*Il6*-R	TGAAGGACTCTGGCTTTGTCT
m*Gdnf*-F	AACATGCCTGGCCTACTTTG
m*Gdnf*-R	GACTTGGGTTTGGGCTATGA
m*Bdnf*-F	GTGACAGTATTAGCGAGTGGG
m*Bdnf*-R	GGGTAGTTCGGCATTGC
m*Nt3*-F	GGTCAGAATTCCAGCCGATGA
m*Nt3*-R	GGCACACACACAGGAAGTGTC
m*Nurr1*-F	CTACGACGTCAAGCCACCTT
m*Nurr1*-R	GGCATCATCTCCTCGGACTG
m*Igf1*-F	TGCTCTTCAGTTCGTGTG
m*Igf1*-R	ACATCTCCAGTCTCCTCAG
m*Igf2*-F	ACAACTTCGATTTGAACCACATTC
m*Igf2*-R	GAGAGCTCAAACCATGCAAACT
m*Gapdh*-F	AGGTCGGTGTGAACGGATTTG
m*Gapdh*-R	TGTAGACCATGTAGTTGAGGT
m*Ccl12*-F	CACGTCCGGAAGCTGAAGAG
m*Ccl12*-R	AGTATGGTCCTGAAGATCACAGC
m*Ccl2*-F	CCACTCACCTGCTGCTACTC
m*Ccl2*-R	AGCTTGGTGACAAAAACTACAGC
m*Cxcl*-F	CCCAAACCGAAGTCATAGCCA
m*Cxcl*-R	CTCCGTTACTTGGGGACACC

Gene name	Sequence (5'->3')
m*Cyba*-F	ACCATGGAGCGATGGTTGT
m*Cyba*-R	AATGGGAGTCCACTGCTCAC
m*Cybb*-F	TTTGTCAAGTGCCCCAAGGT
m*Cybb*-R	ACGCCTATTGTGGTGTTAGGG
m*Cd68*-F	ACGATGACACCTACAGCCAC
m*Cd68*-R	ACAGATATGCCCCAAGCCTTT
m*Sting1*-F	GTCCTGCTAGGTGTCCACTG
m*Sting1*-R	GGAGTATGGCATCAGCAGCC
m*Tnfaip3*-F	AGGGAACTGCCCAGTCTGTA
m*Tnfaip3*-R	TCGTCATTCCAGTTCCGAGT
m*IRF7*-F	GAGCTTGGATCTACTGTGGGC
m*IRF7*-R	CGGCCCTTGTACATGATGGT
m*OAS3*-F	GACCGCTCTAGCATCTCCAC
m*OAS3*-R	TATCGAGTGACAACCTGGCG
m*CASP12*-F	GGCCTGCAGAGGCAGATATAAT
m*CASP12*-R	GCATCTGGGTCAGTTCACCT
m*NAIP2*-F	ACTCACAGATGCGCAGTGAA
m*NAIP2*-R	TCACTTGTGGTTTCCATGGCT
m*NAIP5*-F	TGATTGAAATTGCCAGGGCG
m*NAIP5*-R	CACACATTGACCCAGAGCCT
m*NLRP3*-F	AGCCAGAGTGGAATGACACG
m*NLRP3*-R	TCACCTCTCGGCAGTGGATA
m*CTSB*-F	TGACTGGGGTGATAATGGCTTC
m*CTSB*-R	GAAACTGCAGCACAGAACCG
m*CTSD*-F	GACATCCACTACGGCTCAGG
m*CTSD*-R	TCCACCCTGCGATACCTTGA
m*CTSE*-F	ACTACATCCTGCCGGACTTG
m*CTSE*-R	GTGGGGTGCACTCTTATGCT
m*LGMN*-F	TGGGCTGGAGTAAGTAAGTCTTC
m*LGMN*-R	TTGACGCTGTACCAGTCACC
m*SLC11A1*-F	TGGCTGGGTTCAAACTCCTC
m*SLC11A1*-R	TAGTAGAACTCTGCCCCGCT
m*Gba1*-F	AGGCAGGGCTAGAGAGA
m*Gba1*-R	GAATCTACAGTGGGAAGT

Figure S1

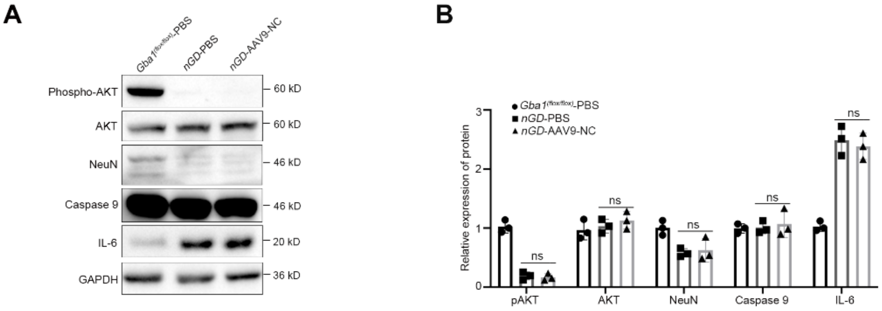

Figure S1. AAV9-NC does not affect *nGD* mouse model.

(A) One month post-delivery, western blot analysis of the whole-brain of *Gba1*$^{(flox/flox)}$-PBS, *nGD*-PBS, and *nGD*-AAV9-NC groups.

(B) Statistical quantitative diagram of (A). ns, no significance.

Figure S2

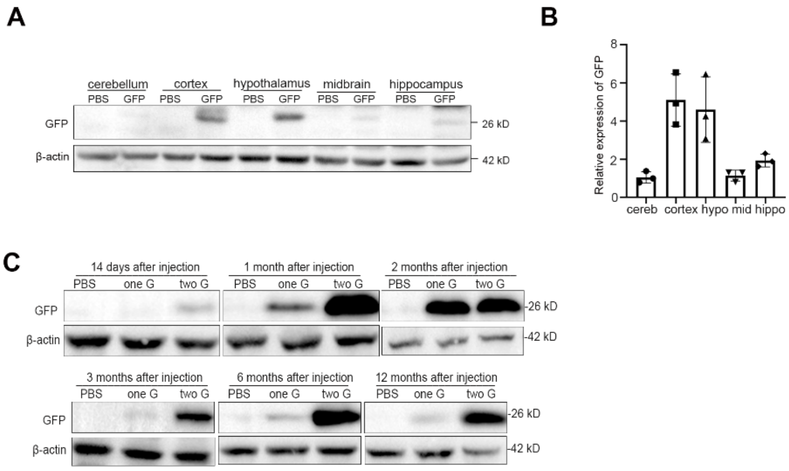

Figure S2. Distribution of AAV9-GFP in the brain of newborn mice after intraparenchymal administration.

(A) Western blot analysis of five brain reigons in C57BL/6 mice one month after AAV9-GFP injection. (B) Statistical quantitative diagram of (A). (C) The dose and time dependence of intraparenchymal administration were detected in C57BL/6 whole-brain.

Figure S3

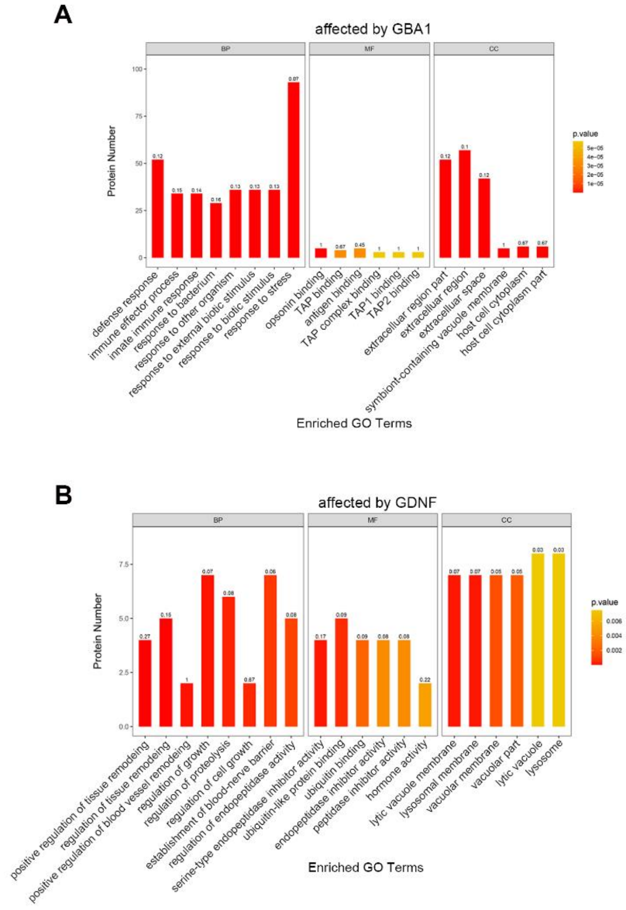

Figure S3. Proteomic detection of *nGD* mice after treatment.

(A) GO functional annotation analysis of proteome sequencing after GBA1 treatment for three months.

(B) GO functional annotation analysis of proteome sequencing after GDNF treatment for three months.

GO, Gene Ontology; BP, Biological Process; MF, Molecular Function; CC, Cellular Compoment.

Figure S4

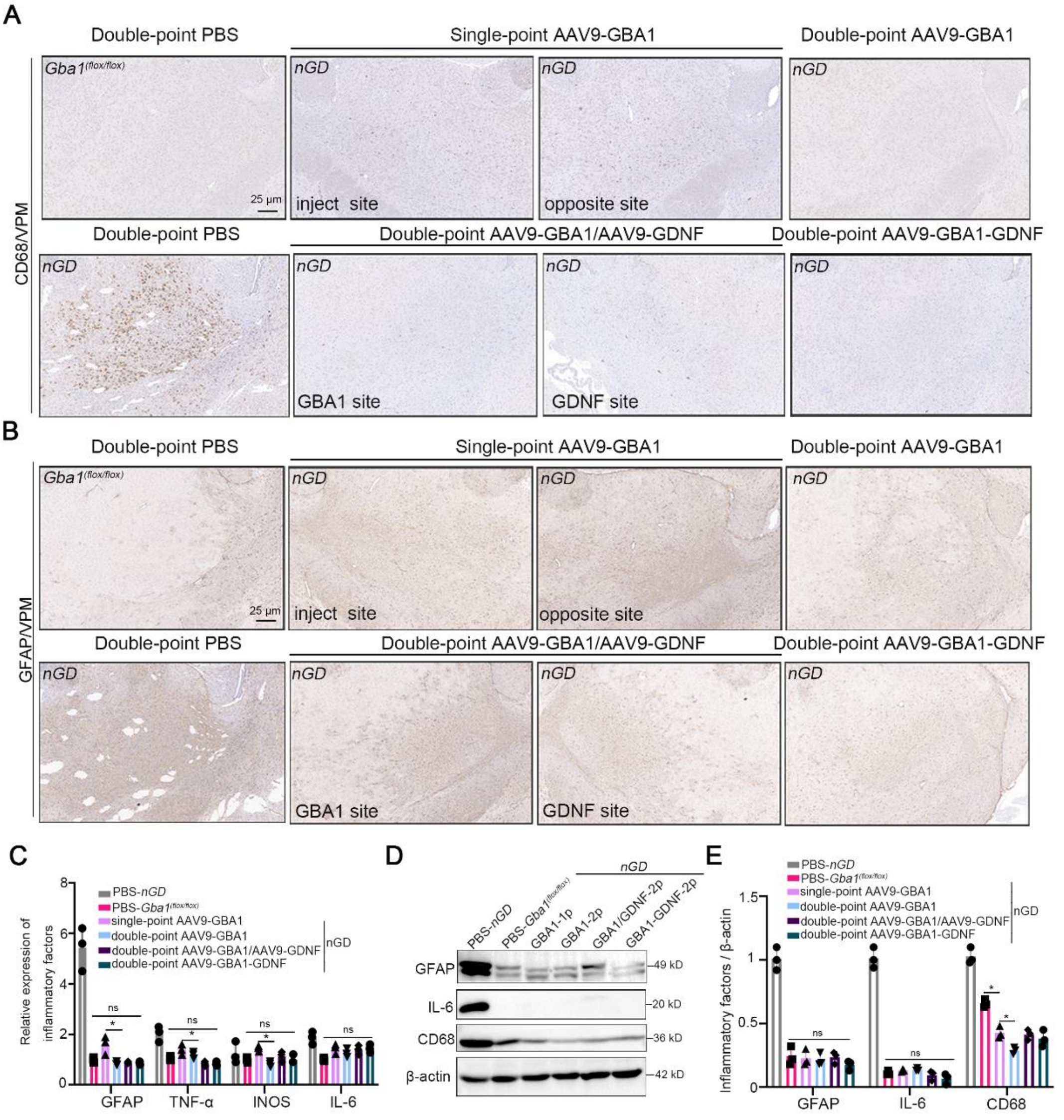

Figure S4. Intracerebral inflammation detection in the *nGD* mice after treatment.

(A) and (B) Three months after treatment, CD68 and GFAP staining was performed in the VPM regions of the mice *Gba1*$^{(flox/flox)}$, *nGD* and *nGD* treatment groups. (C) The mRNA relative expression of inflammatory factors after homogenization in the whole-brain tissue of mice in *Gba1*$^{(flox/flox)}$, *nGD* and *nGD* treatment groups by $2^{-\Delta\Delta CT}$. (D) Western blot was performed to detect the expression levels of inflammation-related proteins in the *Gba1*$^{(flox/flox)}$, *nGD* and *nGD* treatment groups. (E) Quantitative statistical graph of (D). All data are expressed as mean ± standard deviation. The one-way ANOVA method was used for the analysis. Tukey's method was used for multigroup comparisons (n = 3 per group). * $P < 0.05$, ** $P < 0.01$. CD68, Cluster of Differentiation 68. GFAP, Glial fibrillary acidic protein.VPM, ventral posteromedial thalamic nuclei.

Figure S5

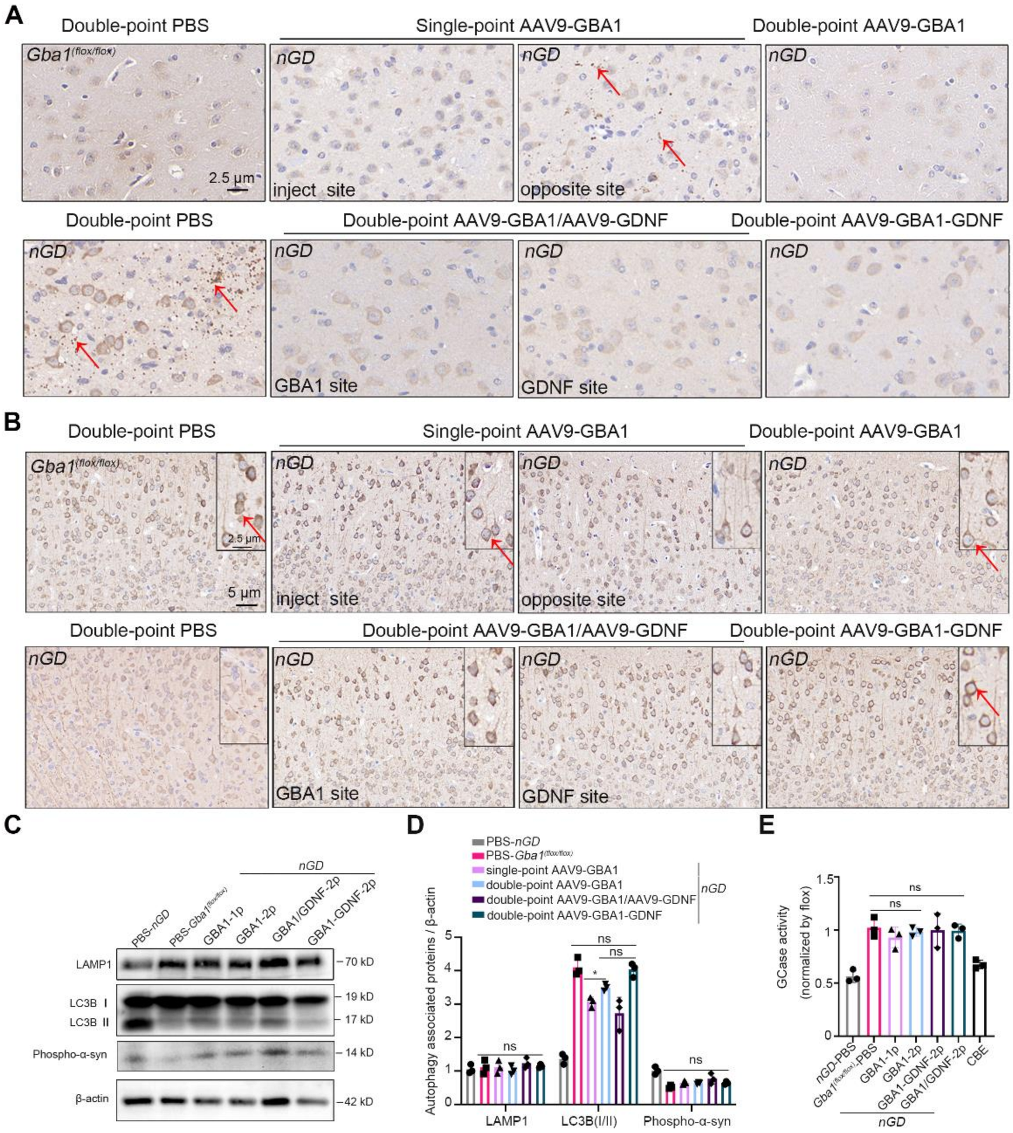

Figure S5. Detecting lysosome function in the *nGD* mice after treatment.

(A) Three months after treatment, LAMP1 staining of lysosomal membrane proteins in VPM sections of mice with *Gba1^(flox/flox)^*, *nGD* and *nGD* treatment groups, with an arrow pointing to positive staining. (B) LC3B staining was performed on sections of the hippocampal S1BF area of mice with *Gba1^(flox/flox)^*, *nGD* and *nGD* treatment groups. The small box indicates an enlarged image of the positive area, and the arrow indicates positive staining. (C) Expression levels of autophagy-related proteins in the *Gba1^(flox/flox)^*, *nGD* and *nGD* treatment groups were detected using Western blot. (D) The statistical quantitative graph of (C). (E) Detection of enzyme activity in mice in the *Gba1^(flox/flox)^*, *nGD* and *nGD* treatment groups after treatment for three months. All data are expressed as mean ± standard deviation. The one-way ANOVA method was used for the analysis. Tukey's method was used for multigroup comparisons (*n* = 3 per group). * $P < 0.05$. VPM, ventral posteromedial thalamic nuclei, SIBF, the primary sensory barrel field.

Figure S6

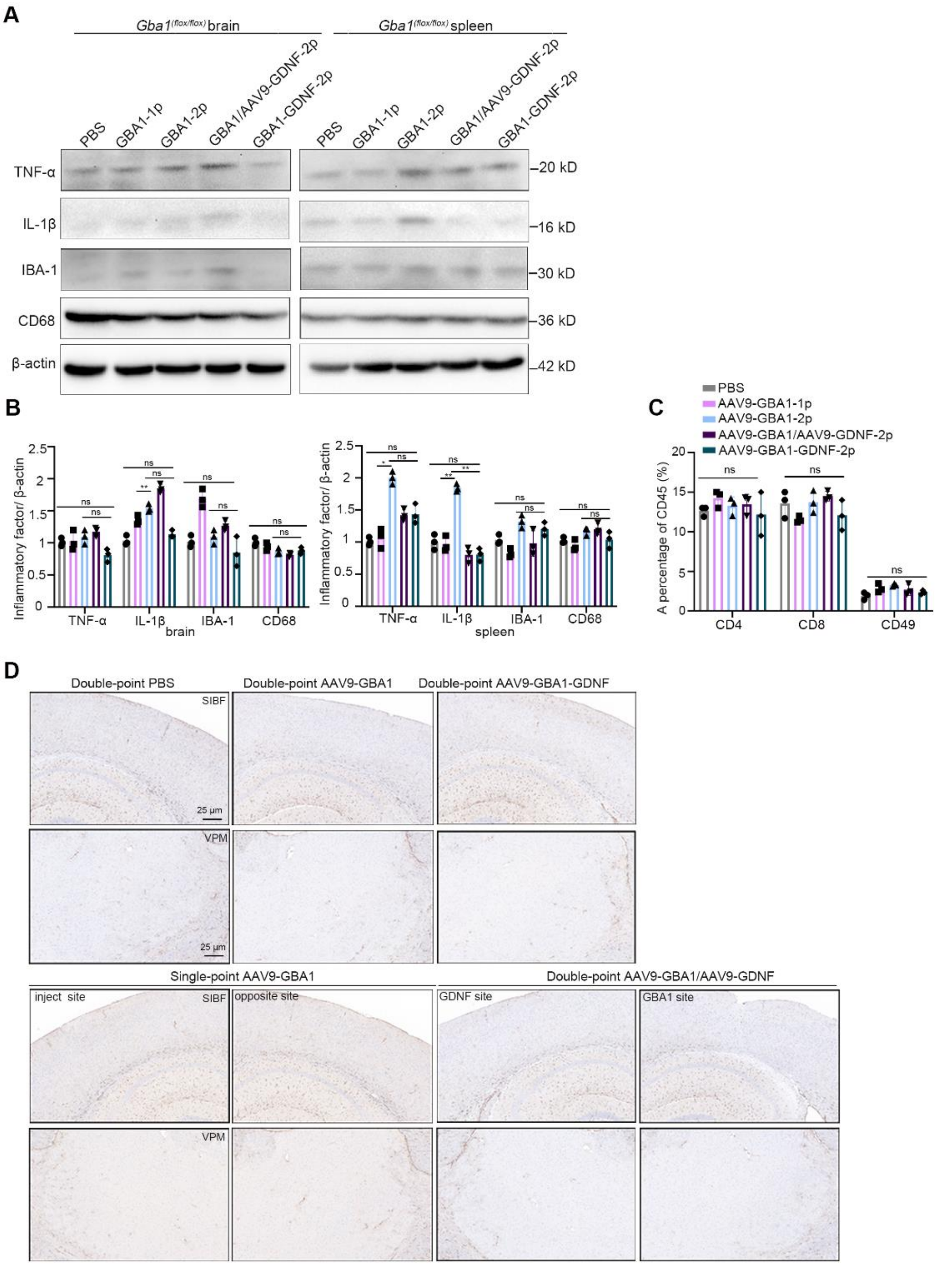

Figure S6. Safety evaluation of intraparenchymal administration of *Gba1*$^{(flox/flox)}$ mice.

(A) Western blot was performed to detect the expression of inflammation-related proteins in *Gba1*$^{(flox/flox)}$ mice 4 months post-intraparenchymal delivery. (B) Diagram of quantitative analysis of A. (C) Flow cytometry was used to detect the percentages of $CD4^{+}$T cells, $CD8^{+}$T cells, and $CD49^{+}$T cells among $CD45^{+}$T cells in *Gba1*$^{(flox/flox)}$ mice 4 months post-intraparenchymal delivery. (D) Positive areas for GFAP were observed in the S1BF and VPM brain sections from *Gba1*$^{(flox/flox)}$ mice 4 months post-intraparenchymal delivery. All data are expressed as mean ± standard deviation. The one-way ANOVA method was used for the analysis. Tukey's method was used for multigroup comparisons (*n* = 3 per group). * $P < 0.05$, ** $P < 0.01$. SIBF, the primary sensory barrel field. VPM, ventral posteromedial thalamic nuclei.

Figure S7

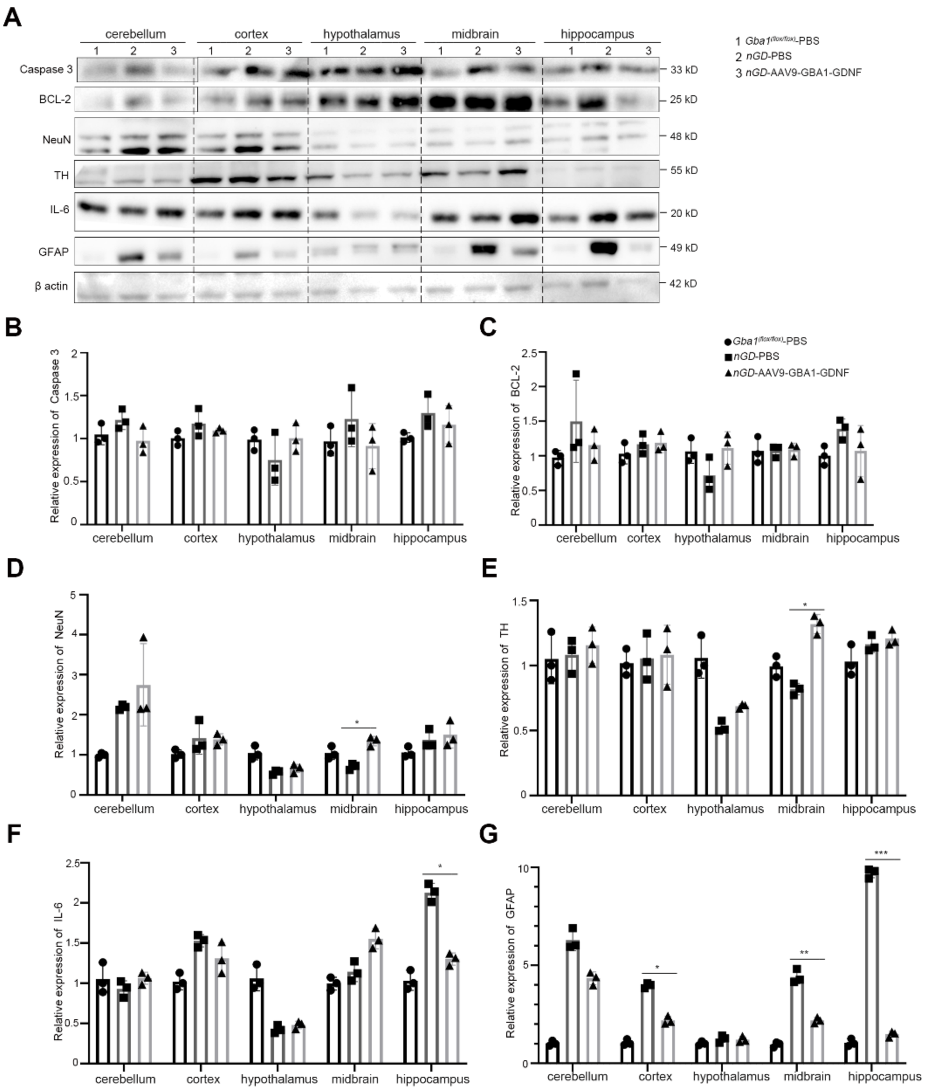

Figure S7. Effect of AAV9-GBA1-GDNF in different brain regions of *nGD* mice.

(A) Western blotting analysis showing the protein level in the cerebellum, cortex , hypothalamus, midbrain and hippocampus of *Gba1*$^{(flox/flox)}$, *nGD*, and *nGD*-AAV9-GBA1-GDNF mice. (B-G) Statistical quantitative diagram of (A). * $P < 0.05$, ** $P < 0.01$.
